# Global, Regional, and National Burden of Tracheal, Bronchial, and Lung Cancer Attributable to Low Fruit Intake From 1990 to 2021

**DOI:** 10.1002/cam4.71584

**Published:** 2026-02-04

**Authors:** Jing‐li Li, Chun‐yi Zhang, Gui‐mei Pu, Ling‐jing Liu, Jian Sun

**Affiliations:** ^1^ Department of Pulmonary and Critical Care Medicine Shaoxing People's Hospital Shaoxing Zhejiang China; ^2^ Department of Pulmonary and Critical Care Medicine The Fourth Affiliated Hospital of Soochow University Suzhou China; ^3^ Department of Pulmonary and Critical Care Medicine The First Affiliated Hospital of Wenzhou Medical University Wenzhou Zhejiang China

**Keywords:** disability‐adjusted life years, estimated annual percentage change, global burden of disease, low fruit intake, lung cancer

## Abstract

**Background:**

Low fruit intake has been identified as a significant modifiable risk factor for tracheal, bronchial, and lung (TBL) cancer. This study aims to quantify the global, regional, and national burden of TBL cancer attributable to low fruit intake from 1990 to 2021.

**Methods:**

Using data from the Global Burden of Disease (GBD) 2021, this descriptive epidemiological study analyzed deaths, disability‐adjusted life years (DALYs), and age‐standardized rates (ASMR and ASDR) attributable to low fruit intake (< 340–350 g/day). Temporal trends were assessed using estimated annual percentage changes (EAPC), and decomposition analyses identified the contributions of aging, population growth, and epidemiological changes to disease burden.

**Results:**

In 2021, low fruit intake caused 66,045 deaths and 1,611,267 DALYs globally, with higher burdens in males. The middle socio‐demographic index (SDI) region recorded the greatest number of deaths and DALYs, while the low‐middle SDI region had the highest ASMR (0.84, 95% uncertainty interval [UI]: 0.44 to 1.18) and ASDR (21.96, 95% UI: 11.39 to 30.93). Temporal trends showed a global decline in ASMR (estimated annual percentage change [EAPC] = −1.89, 95% CI: −1.96 to −1.81) and ASDR (EAPC = −2.23, 95% CI: −2.32 to −2.14) from 1990 to 2021, although increases persisted in some low‐SDI regions. Aging and population growth were major contributors to DALY increases, despite improvements in epidemiological factors.

**Conclusions:**

Low fruit intake significantly contributes to the global TBL cancer burden. Promoting fruit consumption, particularly in low‐SDI regions, is critical for reducing this preventable burden through integrated public health strategies.

## 1 Introduction

Lung cancer remains the leading cause of cancer‐related mortality globally, accounting for an estimated 1.8 million deaths annually as of 2020, representing approximately 18% of total cancer deaths worldwide [1]. Despite advancements in diagnostic techniques and therapeutic options, the burden of lung cancer continues to rise, particularly in low‐ and middle‐income countries [2]. While numerous lifestyle and environmental factors, such as smoking, air pollution, and occupational hazards, have been established as major risk factors for lung cancer development, emerging evidence highlights the significant role of dietary factors, particularly inadequate fruit intake, in increasing lung cancer risk [3].

Fruits are a vital component of a healthy diet, providing essential vitamins, antioxidants, and dietary fiber that collectively reduce oxidative stress and inflammation, both of which are associated with cancer development [4]. The ‘fruit hypothesis’ posits an inverse relationship between fruit intake and the risk of various cancers, including lung cancer [5]. Previous studies have explored this association, but their findings reveal notable limitations. For instance, Wang et al. conducted a meta‐analysis of 32 publications and found a 20% reduction in lung cancer risk with higher fruit intake, with a stronger effect in females; however, the study primarily drew from specific populations and regions, limiting its applicability to a global context and did not address long‐term trends or the broader disease burden [6]. Similarly, Wakai et al. analyzed pooled cohort data from Japan and reported that moderate fruit consumption reduced lung cancer risk among male smokers, yet the findings were restricted to Japanese populations, reducing their generalizability and failed to quantify the attributable impact of low fruit intake [7]. Additionally, Wang et al. performed a dose–response meta‐analysis, identifying a 5% risk reduction per daily fruit serving in current smokers and 4% in former smokers; still, the study relied on observational data without accounting for diverse socio‐demographic settings or the cumulative contribution to lung cancer burden over time [8]. These studies highlight an association between fruit intake and reduced lung cancer risk but fall short of providing a comprehensive, globally relevant analysis.

The Global Burden of Disease (GBD) study offers a robust framework for quantifying the burden of diseases attributable to specific risk factors across countries and regions [9]. However, despite the well‐established link between fruit intake and cancer, few studies have systematically analyzed the burden of lung cancer attributable to low fruit intake on a global scale. Most existing research focuses on individual countries or regions, lacking a comprehensive temporal and spatial analysis [7, 10, 11]. Given the global prevalence of lung cancer and regional disparities in dietary habits, it is crucial to assess the disease burden attributable to low fruit intake using a standardized methodology.

This study aims to address this gap by utilizing data from GBD 2021 to quantify the global, regional, and national burden of lung cancer attributable to low fruit intake from 1990 to 2021. By examining trends over time and across regions, the study seeks to provide actionable insights into how dietary interventions could mitigate the lung cancer burden. The findings are expected to guide policymakers in identifying at‐risk populations and developing public health strategies to promote adequate fruit consumption, ultimately reducing the global incidence of lung cancer.

## 2 Methods

### 2.1 Study Design

This study employed a descriptive epidemiological approach to evaluate the global, regional, and national burden of TBL cancer attributable to low fruit intake from 1990 to 2021. Data were sourced from the Global Burden of Disease (GBD) 2021 study, which provides comprehensive estimates of mortality, morbidity, and disability across 204 countries and territories, as well as for specific risk factors [12]. All data were extracted from the publicly accessible Global Health Data Exchange (GHDx) platform.

### 2.2 Data Sources

The primary dataset for this analysis was the GBD 2021 database, which includes estimates for deaths, disability‐adjusted life years (DALYs), age‐standardized mortality rates (ASMR), and age‐standardized DALY rates (ASDR) attributable to low fruit intake. The GBD study integrates data from a wide range of sources, including vital registration systems, population‐based surveys, national health databases, and epidemiological studies, employing standardized methods to ensure consistency across countries and regions.

### 2.3 Definition of Risk Factor

A diet low in fruit was defined as an average daily consumption of less than 340–350 g, a threshold corresponding to the theoretical minimum risk exposure level (TMREL) established by the Global Burden of Disease (GBD) 2021 Study for optimal health outcomes, based on systematic reviews and meta‐analyses of cohort studies [13]. This definition, standardized across GBD risk factor analyses, includes fresh, frozen, cooked, canned, or dried fruits, but excludes fruit juices—due to their high sugar content and lack of fiber—and salted or pickled fruits—due to their high sodium content and processing effects—to focus on whole fruit consumption. Fruit consumption data were derived from national dietary surveys, food frequency questionnaires, and other nutritional data sources.

### 2.4 Study Population

The methodology for estimating the burden of tracheal, bronchus, and lung (TBL) cancer in the Global Burden of Disease (GBD) 2021 study has been described in detail in previous publications. In summary, the primary input data for TBL cancer estimates were obtained from multiple cancer registries, including Cancer Incidence in Five Continents, NORDCAN, and EUREG. TBL cancer cases were classified according to the International Classification of Diseases (ICD) codes. Specifically, ICD‐10 codes C33‐C34.9, D02.1‐D02.3, D14.2‐D14.3, D38.1, and ICD‐9 codes 162–162.9, 212.2–212.3, 231.1–231.2, and 235.7 were used to define cases of TBL cancer. To generate estimates, the GBD study utilized standardized tools such as the Cause of Death Ensemble Model (CODEm), spatiotemporal Gaussian process regression (ST‐GPR), and the DisMod‐MR model, a Bayesian meta‐regression tool. These methods ensured consistency and comparability in estimating the burden of TBL cancer across regions and time periods. Due to the limited availability of data for individuals under 15 years old in the GBD 2021 dataset, the primary analysis was restricted to individuals aged 15 years and above. Additionally, patients were categorized into 15 age groups, with 5‐year intervals between ages 25 and 90 years, and a final category for those aged ≥ 95 years.

### 2.5 Estimation Process

The comparative risk assessment framework employed by the GBD study was used to estimate the population attributable fraction (PAF) of TBL cancer attributable to low fruit intake. The PAF represents the proportion of the disease burden that could be avoided if the population met recommended fruit intake levels. The PAF was calculated using the following formula:

$$\mathrm{PAF}=p\left(\mathrm{RR}-1\right)/\left[p\left(\mathrm{RR}-1\right)+1\right]$$

where *p* represents the prevalence of low fruit intake, and RR is the relative risk of TBL cancer associated with insufficient fruit consumption, derived from meta‐analyses of cohort studies.

### 2.6 Statistical Analysis

The burden of TBL cancer attributable to low fruit intake was assessed using deaths, DALYs, ASMR, and ASDR across geographic regions, SDI levels, and age groups. DALYs, combining years of life lost (YLLs) and years lived with disability (YLDs), were calculated as follows: YLLs equal deaths multiplied by standard life expectancy at the age of death (from a global reference life table), and YLDs equal TBL cancer cases multiplied by a disability weight (0–1, from GBD surveys) and disease duration, with attributable DALYs derived by applying the population attributable fraction (PAF) to total TBL cancer DALYs. ASMR, expressed per 100,000, was computed by calculating crude mortality rates for each 5‐year age group, weighting these by the GBD standard population, and multiplying the total ASMR by the PAF. Similarly, ASDR (DALYs per 100,000) was determined by summing age‐specific DALY rates, weighting them by the standard population, and applying the PAF. Age‐standardized rates were calculated using the direct standardization method, applying age‐specific rates to the GBD global standard population: $\mathrm{ASR}=\sum \left(\mathrm{ri}\times \mathrm{wi}\right)$.Where is the age‐specific rate for the i th age group, and wi is the proportion of the standard population in the corresponding age group. The estimated annual percentage change (EAPC) was calculated to evaluate the temporal trends in ASMR and ASDR over the specified period. The regression model *y* = *α* + *βx* + *ε* was constructed, where *x* represents the calendar year and *y* = ln (ASMR) or ln (ASDR), *α* is the intercept, *β* is the slope, and *ε* is the error term. The EAPC and its 95% confidence interval (CI) were derived using the formula 100 × (exp(β) − 1). An EAPC with its 95% CI > 0 indicated an increasing trend, whereas an EAPC with its 95% CI < 0 indicated a decreasing trend. If the 95% CI included 0, the ASMR or ASDR was considered stable over time. Temporal trends from 1990 to 2021 were further analyzed using Joinpoint regression to estimate the average annual percentage change (AAPC) and its corresponding 95% CIs. An age‐period‐cohort (APC) analysis was conducted to explore the contributions of age, period, and cohort effects on TBL cancer trends [14]. Additionally, the Bayesian Age‐Period‐Cohort (BAPC) model was employed to project the burden from 2022 to 2035. Unlike classical APC models, BAPC models do not rely on parametric assumptions, providing more robust forecasts, as demonstrated by Bray and colleagues [15]. A decomposition analysis was performed to determine the contributions of population growth, aging, and epidemiological changes to the burden of TBL cancer across different SDI levels, identifying key drivers of disease burden at varying stages of development. To quantify SDI‐related inequalities, the slope index of inequality (SII) and the concentration index (CI) were used [16]. The SII was calculated by regressing country‐level disease prevalence against a sociodemographic development‐related position scale, defined by the midpoint of the cumulative population distribution ranked by SDI [17]. The CI was derived from the Lorenz concentration curve, representing the cumulative distribution of population rank versus disease prevalence, and computed by integrating the area under the curve [18]. All statistical analyses were conducted using R statistical software (version 5.0.4) and the Joinpoint Regression Program (version 4.7). A *p*‐value of less than 0.05 was considered statistically significant. As this study utilized publicly available, de‐identified data from the GBD database, no ethical approval or informed consent was required.

## 3 Results

### 3.1 Global Burden of TBL Cancer Attributable to Diet Low in Fruit in 2021

In 2021, the total deaths of TBL cancer attributable to diet low in fruit were 66,045 (95% UI: 34,005–97,033), with 44,328 (95% UI: 22,872–64,126) cases in males and 21,717 (95% UI: 11,100–32,326) in females. The number of DALYs reached 1,611,267 (95% UI: 828,053–2,347,369), with 1,097,919 (95% UI: 566,432–1,597,498) in males and 513,347 (95% UI: 261,758–754,495) in females. The ASMR for both sexes combined was 0.77 (95% UI: 0.40–1.13) per 100,000 population, with 1.12 (95% UI: 0.58–1.63) for males and 0.47 (95% UI: 0.24–0.70) for females. Similarly, the ASDR was 18.46 (95% UI: 9.49–26.90) per 100,000 for both sexes, 26.48 (95% UI: 13.66–38.50) in males, and 11.26 (95% UI: 5.74–16.55) in females (Table 1).

**TABLE 1:** Global and regional deaths and DALYs of TBL cancer attributable to diet low in fruit in 1990 and 2021, and the EAPC from 1990 to 2021.

Location	1990				2021				EAPC (1990–2021)	
	Deaths cases	ASMR per 100,000	DALYs	ASDR per 100,000	Deaths cases	ASMR per 100,000	DALYs	ASDR per 100,000	ASMR	ASDR
	No. (95% UI)	No. (95% UI)	No. (95% UI)	No. (95% UI)	No. (95% UI)	No. (95% UI)	No. (95% UI)	No. (95% UI)	No. (95% CI)	No. (95% CI)
Global	51621.37 (25769.75, 75860.15)	1.30 (0.65, 1.91)	1435375.14 (721498.80, 2119984.28)	34.39 (17.24, 50.70)	66045.46 (34005.89, 97033.46)	0.77 (0.40, 1.13)	1611267.04 (828053.56, 2347369.49)	18.46 (9.49, 26.90)	−1.89 (−1.96, −1.81)	−2.23 (−2.32, −2.14)
*Sex*										
Male	37726.67 (18866.62, 56539.44)	2.08 (1.05, 3.11)	1059478.50 (534061.42, 1599531.77)	53.64 (27.01, 80.74)	44328.29 (22872.11, 64126.67)	1.12 (0.58, 1.63)	1097919.60 (566432.74, 1597498.78)	26.48 (13.66, 38.50)	−2.15 (−2.21, −2.09)	−2.47 (−2.54, −2.39)
Female	13894.70 (7255.52, 20690.59)	0.65 (0.34, 0.97)	375896.64 (196209.09, 560465.64)	17.22 (8.98, 25.64)	21717.17 (11100.42, 32326.79)	0.47 (0.24, 0.70)	513347.43 (261758.99, 754495.90)	11.26 (5.74, 16.55)	−1.32 (−1.42, −1.22)	−1.64 (−1.76, −1.53)
*SDI regions*										
High SDI	13872.00 (7056.80, 20227.43)	1.25 (0.64, 1.82)	336402.13 (170223.99, 487385.34)	31.18 (15.77, 45.16)	16064.47 (8039.53, 24167.47)	0.73 (0.37, 1.10)	322708.77 (163061.57, 479678.81)	16.18 (8.21, 24.09)	−1.64 (−1.71 −1.56)	−2.03 (−2.12 −1.95)
High‐middle SDI	15652.67 (7764.87, 23387.83)	1.55 (0.77, 2.31)	451238.24 (225372.62, 672449.64)	43.32 (21.63, 64.65)	14174.69 (7071.55, 21347.38)	0.71 (0.36, 1.07)	338956.38 (167188.23, 513037.21)	17.17 (8.48, 25.96)	−2.93 (−3.11 −2.75)	−3.46 (−3.65 −3.27)
Middle SDI	14842.70 (7433.52, 22198.23)	1.44 (0.72, 2.15)	435241.92 (214869.03, 649544.74)	37.98 (18.93, 56.70)	20847.49 (10804.29, 30841.29)	0.79 (0.41, 1.17)	529165.89 (271427.98, 776882.84)	18.97 (9.76, 27.83)	−2.21 (−2.31 −2.10)	−2.53 (−2.65 −2.41)
Low‐middle SDI	5794.91 (3055.61, 8625.02)	0.94 (0.50, 1.41)	169941.35 (89659.36, 252673.31)	25.21 (13.29, 37.46)	12105.29 (6258.33, 17014.52)	0.84 (0.44, 1.18)	339645.21 (176183.28, 478077.58)	21.96 (11.38, 30.93)	−0.42 (−0.54 −0.31)	−0.47 (−0.59 −0.36)
Low SDI	1409.76 (744.02, 2181.23)	0.62 (0.33, 0.96)	41193.83 (21754.85, 63799.66)	16.45 (8.68, 25.52)	2800.08 (1383.32, 3997.61)	0.57 (0.28, 0.82)	79513.10 (39178.95, 113065.90)	14.28 (7.06, 20.35)	−0.35 (−0.46 −0.24)	−0.57 (−0.68 −0.47)
*GBD regions*										
Andean Latin America	78.43 (39.65, 122.29)	0.40 (0.20, 0.62)	2084.74 (1060.79, 3221.80)	9.59 (4.86, 14.87)	128.37 (61.86, 200.19)	0.22 (0.11, 0.34)	3053.10 (1460.15, 4756.15)	5.06 (2.42, 7.86)	−2.10 (−2.36, −1.84)	−2.33 (−2.63, −2.02)
Australasia	240.12 (119.86, 350.58)	1.01 (0.50, 1.47)	5697.55 (2861.23, 8319.46)	24.37 (12.22, 35.55)	328.70 (168.98, 487.59)	0.59 (0.31, 0.88)	6699.61 (3486.33, 9909.47)	13.06 (6.83, 19.32)	−1.71 (−1.76, −1.66)	−2.00 (−2.07, −1.93)
Caribbean	124.28 (61.78, 185.51)	0.49 (0.24, 0.72)	3071.37 (1528.78, 4605.88)	11.61 (5.77, 17.42)	167.05 (80.68, 247.90)	0.31 (0.15, 0.46)	3953.66 (1895.85, 5867.19)	7.35 (3.52, 10.89)	−1.42 (−1.53, −1.32)	−1.51 (−1.61, −1.41)
Central Asia	859.60 (438.46, 1252.09)	1.78 (0.90, 2.59)	26223.16 (13391.42, 38103.33)	52.05 (26.47, 75.81)	381.08 (196.46, 559.65)	0.46 (0.24, 0.68)	10853.60 (5571.65, 15980.34)	12.13 (6.24, 17.81)	−4.75 (−4.97, −4.53)	−5.16 (−5.39, −4.92)
Central Europe	2074.49 (1054.19, 3002.17)	1.37 (0.70, 1.99)	58378.24 (29834.89, 84084.65)	38.55 (19.69, 55.54)	2223.53 (1135.99, 3283.08)	1.00 (0.51, 1.47)	51738.42 (26282.77, 76270.88)	24.62 (12.50, 36.26)	−1.20 (−1.42, −0.98)	−1.63 (−1.87, −1.39)
Central Latin America	252.14 (126.81, 366.41)	0.32 (0.16, 0.46)	6687.26 (3362.16, 9669.58)	7.56 (3.80, 10.97)	483.46 (243.80, 710.97)	0.20 (0.10, 0.29)	11772.24 (5938.96, 17378.16)	4.62 (2.33, 6.82)	−1.71 (−1.79, −1.64)	−1.77 (−1.85, −1.69)
Central Sub‐Saharan Africa	86.46 (39.29, 152.22)	0.39 (0.18, 0.68)	2584.18 (1183.38, 4532.75)	10.18 (4.63, 17.96)	251.66 (100.60, 466.00)	0.46 (0.19, 0.84)	7603.64 (3019.61, 14241.55)	11.94 (4.78, 22.04)	0.43 (0.29, 0.57)	0.38 (0.24, 0.52)
East Asia	17899.99 (8752.23, 27450.77)	2.12 (1.04, 3.22)	520775.78 (255283.07, 780491.09)	54.78 (26.76, 82.71)	19553.39 (9988.82, 30556.80)	0.92 (0.47, 1.44)	455283.76 (229549.26, 716373.34)	20.72 (10.48, 32.50)	−2.99 (−3.16, −2.82)	−3.49 (−3.66, −3.32)
Eastern Europe	5642.04 (2927.66, 8218.47)	1.97 (1.02, 2.87)	164166.26 (85551.40, 238346.15)	57.59 (30.06, 83.50)	2825.95 (1449.49, 4147.27)	0.80 (0.41, 1.17)	72401.66 (37070.44, 105901.21)	21.24 (10.90, 31.11)	−3.60 (−3.87, −3.32)	−3.96 (−4.26, −3.67)
Eastern Sub‐Saharan Africa	692.86 (363.25, 1065.38)	0.91 (0.48, 1.40)	20325.02 (10574.70, 31505.51)	24.45 (12.77, 37.66)	1025.18 (522.30, 1468.28)	0.65 (0.33, 0.93)	28946.99 (14805.10, 41514.40)	15.65 (7.97, 22.36)	−1.30 (−1.44, −1.16)	−1.70 (−1.86, −1.55)
High‐income Asia Pacific	2045.27 (1054.21, 2952.98)	1.04 (0.54, 1.49)	47430.45 (24351.48, 68656.75)	23.25 (11.95, 33.66)	3788.85 (1927.38, 5598.52)	0.72 (0.36, 1.06)	65117.03 (32978.25, 96289.81)	15.02 (7.65, 22.08)	−1.00 (−1.20, −0.79)	−1.15 (−1.39, −0.90)
High‐income North America	4797.84 (2456.91, 7008.65)	1.37 (0.70, 2.01)	116402.99 (59213.30, 170119.53)	34.99 (17.78, 50.97)	4753.87 (2371.77, 7308.42)	0.70 (0.35, 1.07)	98586.44 (49205.03, 150589.69)	15.31 (7.67, 23.30)	−2.13 (−2.21, −2.06)	−2.62 (−2.70, −2.55)
North Africa and Middle East	464.78 (228.43, 726.32)	0.28 (0.14, 0.44)	13430.08 (6595.65, 21037.66)	7.25 (3.56, 11.35)	844.81 (424.82, 1277.11)	0.19 (0.10, 0.29)	23505.73 (11719.35, 35334.63)	4.70 (2.36, 7.10)	−1.44 (−1.58, −1.30)	−1.62 (−1.75, −1.48)
Oceania	26.13 (11.91, 44.32)	0.95 (0.44, 1.63)	762.29 (346.10, 1300.83)	23.56 (10.70, 40.04)	57.02 (25.93, 96.17)	0.82 (0.38, 1.36)	1643.02 (735.44, 2810.26)	19.92 (9.07, 33.59)	−0.41 (−0.49, −0.33)	−0.46 (−0.54, −0.38)
South Asia	6186.05 (3329.67, 9062.93)	1.06 (0.57, 1.56)	185148.31 (99645.07, 270387.56)	28.61 (15.39, 41.85)	16739.22 (8630.71, 23927.20)	1.12 (0.58, 1.61)	469725.09 (241738.98, 673435.24)	29.62 (15.24, 42.45)	0.10 (−0.01, 0.21)	0.03 (−0.08, 0.14)
Southeast Asia	3462.44 (1761.77, 5213.15)	1.38 (0.70, 2.07)	99019.98 (50496.87, 149303.27)	35.60 (18.14, 53.73)	4374.16 (2127.22, 6611.79)	0.68 (0.33, 1.03)	118213.87 (57829.34, 179100.18)	16.87 (8.24, 25.48)	−2.53 (−2.65, −2.41)	−2.67 (−2.81, −2.53)
Southern Latin America	326.23 (164.36, 475.61)	0.70 (0.35, 1.03)	8790.76 (4446.48, 12844.04)	18.75 (9.49, 27.41)	279.92 (142.19, 421.19)	0.32 (0.16, 0.48)	6388.76 (3226.03, 9596.85)	7.47 (3.75, 11.23)	−2.55 (−2.69, −2.41)	−3.00 (−3.14, −2.85)
Southern Sub‐Saharan Africa	462.63 (242.81, 701.08)	1.70 (0.90, 2.58)	13674.30 (7150.69, 20679.83)	46.05 (24.07, 69.57)	1067.82 (542.64, 1556.32)	1.86 (0.95, 2.68)	30227.94 (15316.89, 43759.08)	48.06 (24.40, 69.84)	0.20 (−0.16, 0.57)	0.06 (−0.32, 0.44)
Tropical Latin America	322.21 (162.97, 475.26)	0.36 (0.18, 0.54)	8993.12 (4514.61, 13360.05)	9.14 (4.60, 13.56)	576.37 (296.17, 858.95)	0.23 (0.12, 0.34)	13945.02 (7176.54, 20904.29)	5.34 (2.76, 8.01)	−1.63 (−1.72, −1.54)	−1.93 (−2.02, −1.84)
Western Europe	5340.42 (2698.58, 7762.14)	0.92 (0.47, 1.34)	125376.02 (63353.47, 181484.93)	22.91 (11.58, 33.11)	5650.38 (2848.59, 8259.26)	0.60 (0.30, 0.87)	116950.48 (59350.07, 169417.31)	13.97 (7.06, 20.24)	−1.21 (−1.30, −1.12)	−1.42 (−1.49, −1.34)
Western Sub‐Saharan Africa	236.96 (119.49, 352.28)	0.28 (0.14, 0.42)	6353.30 (3193.60, 9375.72)	6.85 (3.45, 10.14)	544.65 (267.32, 815.09)	0.30 (0.15, 0.45)	14656.99 (7088.00, 22043.05)	7.01 (3.43, 10.46)	0.45 (0.34, 0.56)	0.30 (0.20, 0.41)

*Note:* The rates are reported per 100,000 people per year. Data in parentheses are 95% uncertainty intervals for cases and age‐standardized rates of deaths and DALYs, and 95% confidence intervals for EAPCs.

Abbreviations: ASDR, age‐standardized DALYs rate; ASMR, age‐standardized mortality rate; CI, confidence interval; DALYs, disability‐adjusted life‐years; EAPC, estimated annual percentage change; SDI, socio demographic index; UI, uncertainty interval.

At the SDI level, the middle SDI region recorded the greatest number of deaths and DALYs in 2021, while the highest ASMR and ASDR were observed in the low–middle SDI region (Table 1).

At the GBD regional level, Southern Sub‐Saharan Africa had the greatest burden in 2021, with an ASMR of 1.86 (95% UI: 0.95–2.68) and an ASDR of 48.06 (95% UI: 24.40–69.84) per 100,000 population. Most regions experienced declining trends in ASMR and ASDR, with Central Asia and Eastern Europe showing the fastest declines. However, four regions—Central Sub‐Saharan Africa, Western Sub‐Saharan Africa, Southern Sub‐Saharan Africa, and South Asia—exhibited year‐on‐year increases in ASMR and ASDR (Table 1).

**FIGURE 1:**
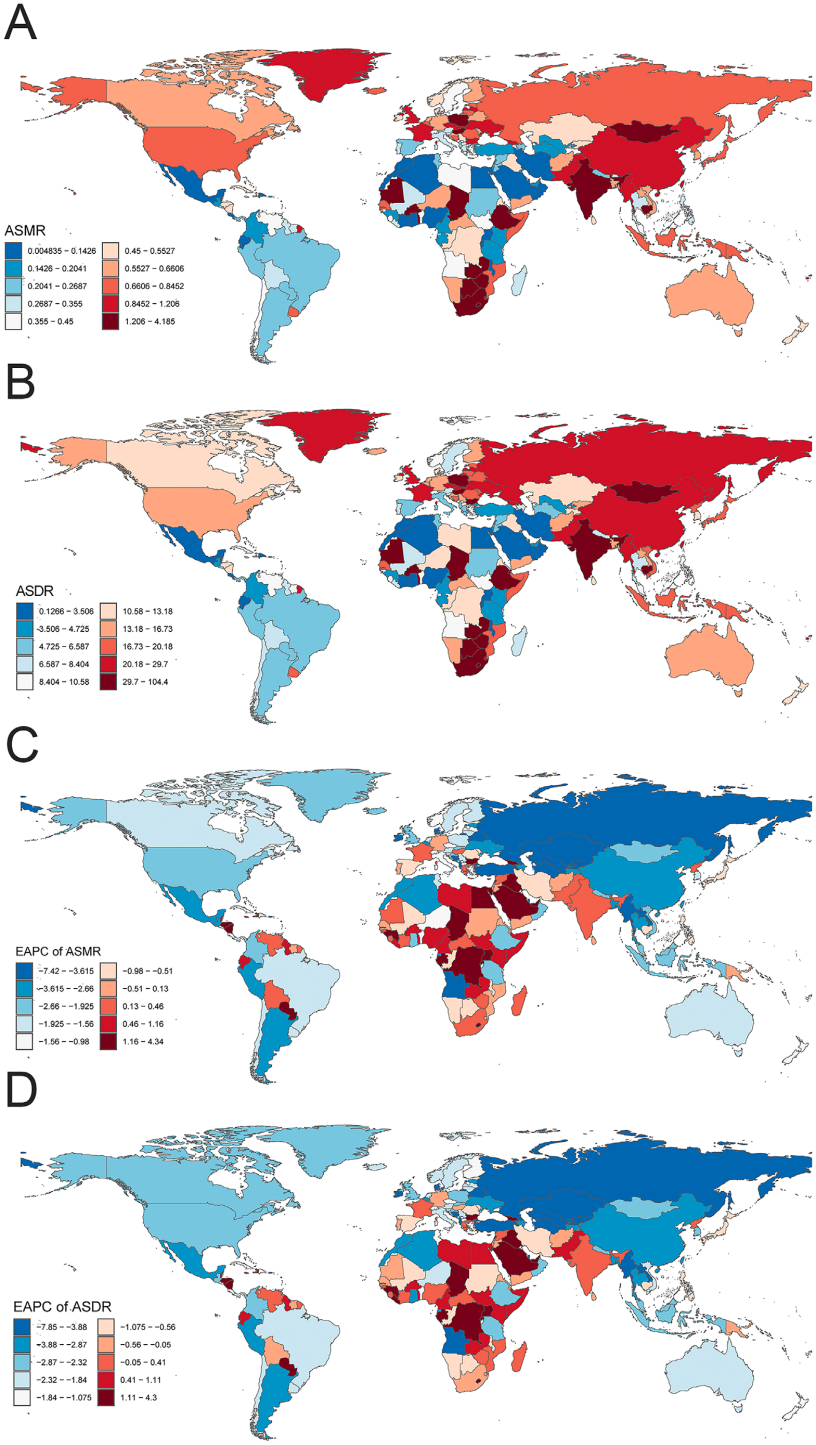
The spatial distribution of ASMR (A) and ASDR (B) in TBL cancer attributable to diet low in fruit in 2021, and the EAPC of ASMR (C) and ASDR (D) in TBL cancer attributable to diet low in fruit. ASDR, age‐standardized DALYs rate; ASMR, age‐standardized mortality rate; DALYs, disability‐adjusted life years; EAPC, estimated annual percentage change; SDI, socio‐demographic index; TBL, tracheal, bronchus, and lung.

At the national level, substantial heterogeneity was observed across 204 countries. Mongolia had the highest ASMR at 4.19 (95% UI: 2.09–6.34) and ASDR at 104.40 (95% UI: 51.28–155.72), whereas Rwanda had the lowest values (ASMR = 0.005, 95% UI: 0.002–0.010; ASDR = 0.13, 95% UI: 0.05–0.27) (Figure 1A,B; Tables S1 and S2). The fastest increase in ASMR and ASDR was observed in Lebanon, with EAPCs 4.34 (95% CI: 3.63–5.05) and 4.30 (95% CI: 3.60–5.01), respectively. Conversely, Kazakhstan showed the largest decrease in ASMR (EAPC = −7.42, 95% CI: −7.81 to −7.02) and ASDR (EAPC = −7.85, 95% CI: −8.26 to −7.42) (Figure 1C,D; Tables S3 and S4).

**FIGURE 2:**
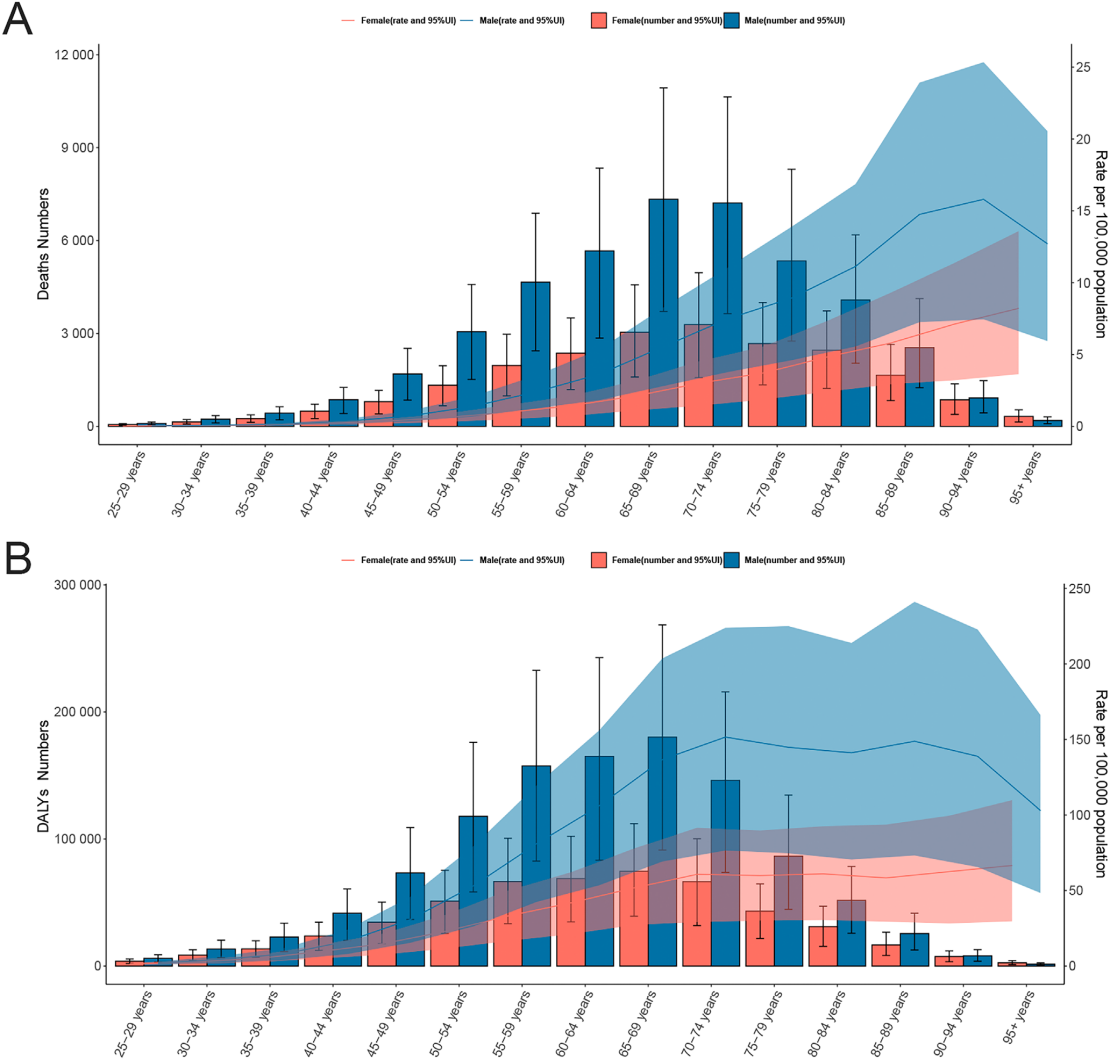
The number of deaths and ASMR for TBL cancer attributable to diet low in fruit globally, stratified by sex and age group in 2021 (A). The number of DALYs and ASDR for TBL cancer attributable to diet low in fruit globally, stratified by sex and age group (B). ASDR, age‐standardized DALYs rate; ASMR, age‐standardized mortality rate; DALYs, disability‐adjusted life years; SDI, socio‐demographic index; TBL, tracheal, bronchus, and lung; UI, uncertainty interval.

Further analysis by age and sex showed that in 2021, male patients had consistently higher numbers and rates of TBL cancer deaths and DALYs than females. Deaths peaked at age 65–69 years for males and 70–74 years for females, while DALYs peaked at 65–69 years in both sexes. The highest death rate was recorded at 90–94 years in males and ≥ 95 years in females, while the DALY rate peaked at 70–74 years in males and ≥ 95 years in females (Figure 2; Tables S5 and S6).

### 3.2 Temporal Trends in TBL Cancer Burden Attributable to Diet Low in Fruit

**FIGURE 3:**
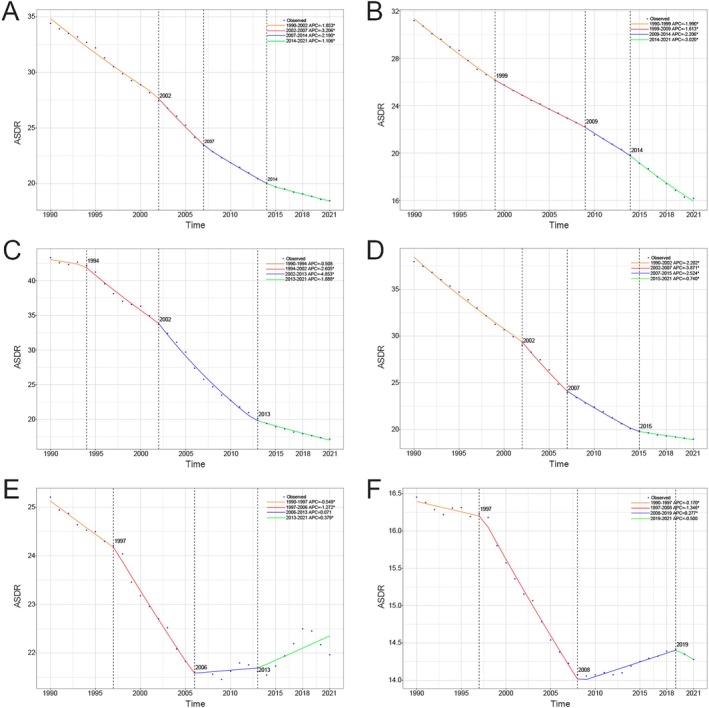
Temporal trends of TBL cancer ASDR attributable to diet low in fruit from 1990 to 2021 globally and across five SDI regions. (A) APPC of ASDR globally; (B) APPC of ASDR in high SDI regions; (C) APPC of ASDR in high‐middle SDI regions; (D) APPC of ASDR in middle SDI regions; (E) APPC of ASDR in low‐middle SDI regions; (F) APPC of ASDR in low SDI regions. AAPC, average annual percentage change; ASDR, age‐standardized DALYs rate; DALYs, disability‐adjusted life years; SDI, socio‐demographic index; TBL, tracheal, bronchus, and lung.

From 1990 to 2021, the ASDR of TBL cancer burden attributable to diet low in fruit globally and across the five SDI regions demonstrated significantly decreasing trends (Global: AAPC = −0.514; 95% CI: −0.525 to −0.503; high SDI regions: AAPC = −0.488; 95% CI: −0.496 to −0.480; high‐middle SDI regions: AAPC = −0.855; 95% CI: −0.884 to −0.826; middle SDI regions: AAPC = −0.626; 95% CI: −0.632 to −0.619; low‐middle SDI regions: AAPC = −0.102; 95% CI: −0.114 to −0.090; low SDI regions: AAPC = −0.068; 95% CI: −0.073 to −0.064). Globally, the TBL cancer burden attributable to diet low in fruit exhibited a significant decline, with a gradual decrease from 1990 to 2002 (annual percentage change [APC] = −1.853), followed by an accelerated decline from 2002 to 2007 (APC = −3.206) and from 2007 to 2014 (APC = −2.190), and subsequently, a slowdown in the rate of decline from 2014 to 2021 (APC = −1.106). Except for the low‐middle SDI and low SDI regions, all regions showed a pattern of initial acceleration followed by deceleration in the decline rate. Specifically, the low‐middle SDI regions experienced a decrease from 1990 to 1997 (APC = −0.549) and from 1997 to 2006 (APC = −1.272), followed by persistent increases in later periods: from 2006 to 2013 (APC = 0.071), and from 2013 to 2021 (APC = 0.379). Similarly, the low SDI regions saw an initial decrease from 1990 to 1997 (APC = −0.170) and 1997 to 2006 (AP C = −1.346), followed by an increase from 2008 to 2019 (APC = 0.277), and another decline from 2019 to 2021 (APC = −0.500) (Figure 3).

### 3.3 The Correlation Between the SDI and ASR, the EAPC and ASR, and SDI

**FIGURE 4:**
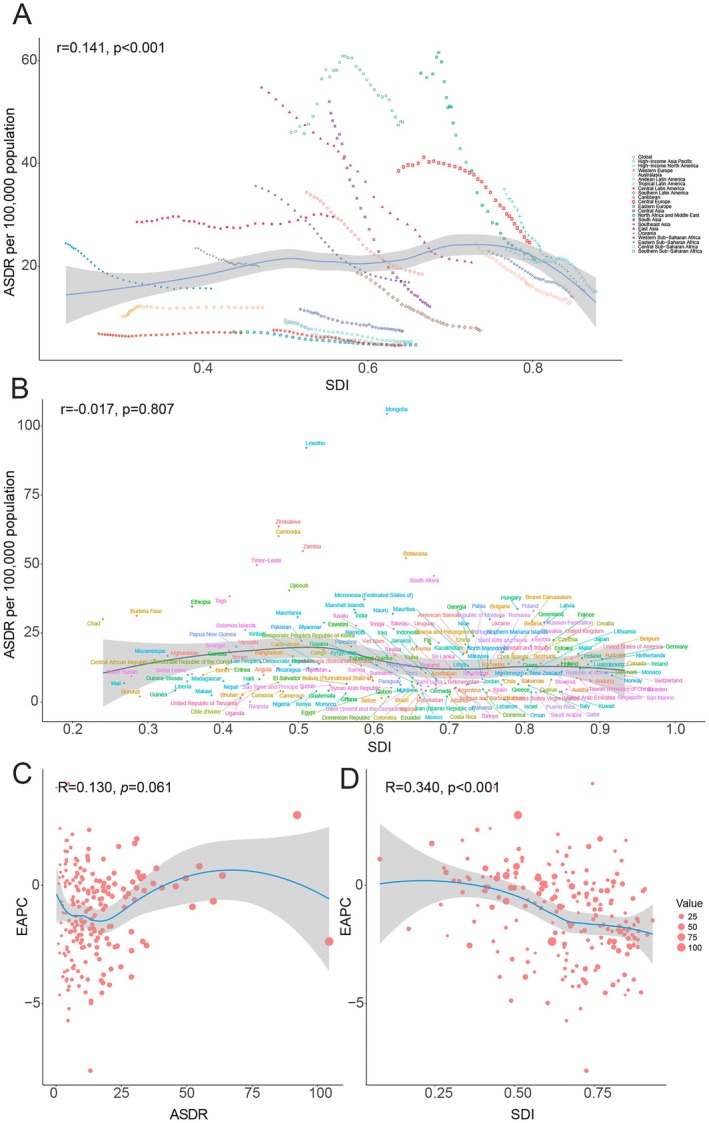
The correlation between TBL cancer attributable to diet low in fruit in ASDR and SDI at the regional (A) and national (B) levels between 1990 and 2021. The correlation between EAPC of ASDR and ASDR in 1990 (C) and EAPC of ASDR and SDI in 2021 (D). The size of each dot corresponds to the DALYs of TBL cancer attributable to diet low in fruit. The *R* and *p*‐values were derived from Pearson correlation analysis. ASDR, age‐standardized DALYs rate; DALYs, disability‐adjusted life years; EAPC, estimated annual percentage change; SDI, socio‐demographic index; TBL, tracheal, bronchus, and lung.

We further explored the correlations between SDI and ASR, as well as between EAPC and SDI or ASR, with the results summarized in Figure 4 and Figure S1. At the regional level, a positive correlation was observed between SDI and ASDR (*r* = 0.141, *p* < 0.001) (Figure 4B). In certain regions, such as South Asia, Southern Sub–Saharan Africa, and Central Europe, ASDR levels were higher than predicted. However, at the national level, no significant association between SDI and ASDR was found (r = −0.017, *p* = 0.807) (Figure 4B). Additionally, EAPC of ASDR was not significantly correlated with ASDR (*r* = 0.130, *p* = 0.061) (Figure 4C). In contrast, a positive correlation was found between EAPC of ASDR and SDI (r = 0.340, *p* < 0.001) (Figure 4D), indicating that the burden of TBL cancer attributable to diet low in fruit increased more rapidly in countries with high SDI levels. Comparable patterns were observed for ASMR and its corresponding correlations with SDI and EAPC, as shown in Figure S1.

### 3.4 Age, Period and Cohort Effects on TBL Cancer Burden Attributable to Diet Low in Fruit

**FIGURE 5:**
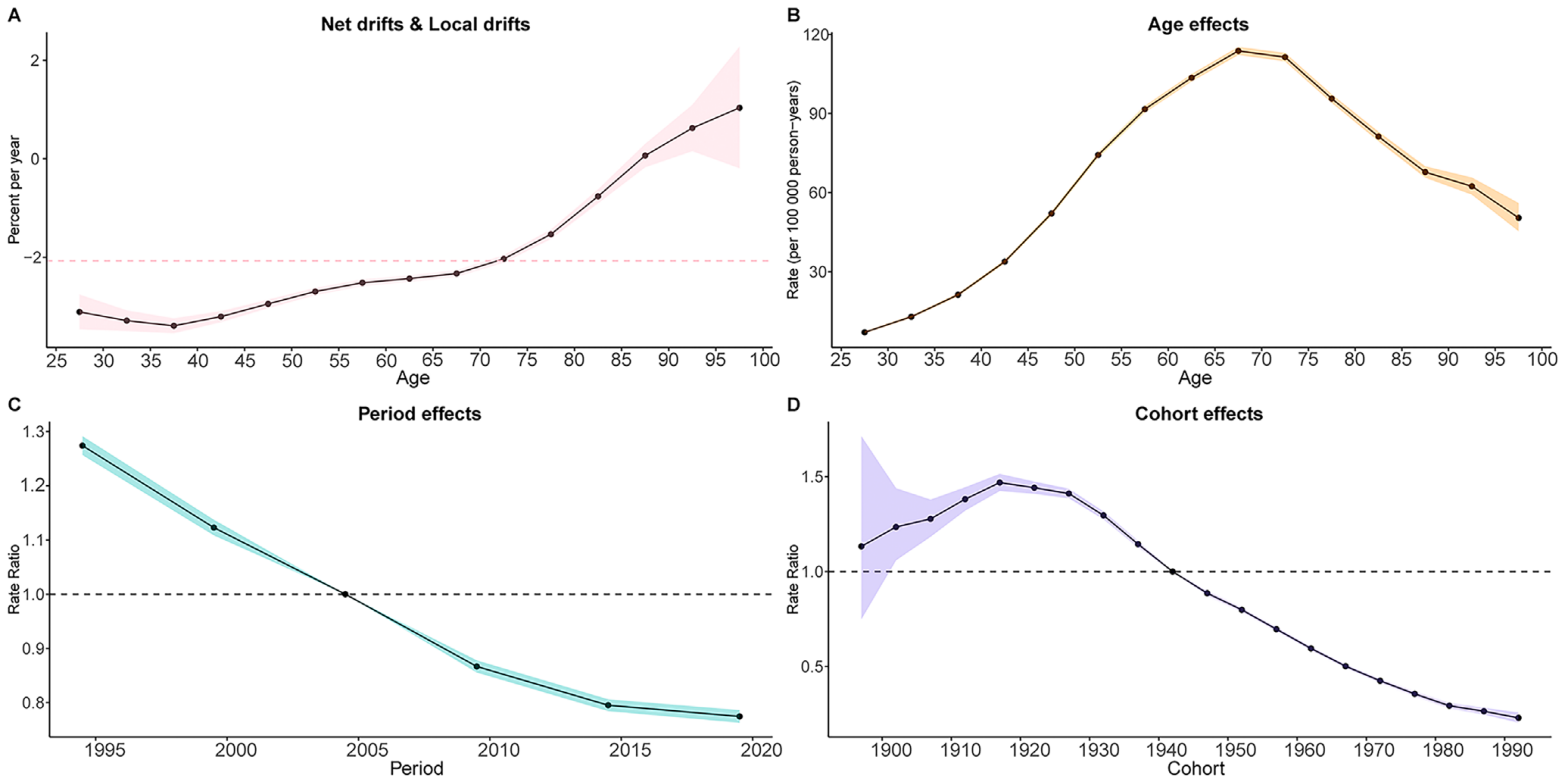
Local drifts of TBL cancer DALYs attributable to diet low in fruit globally (A). Age‐period‐cohort analysis of TBL cancer DALYs attributable to diet low in fruit globally from 1990 to 2021, showing age (B), period (C), and cohort (D) effects. DALYs, disability‐adjusted life years; TBL, tracheal, bronchus, and lung.

The results of the age–period–cohort (APC) analysis for DALY and death rates of TBL cancer attributable to low fruit intake from 1990 to 2021 are shown in Figure 5 and Figure S2. According to the Wald chi‐square tests, the age, period, and cohort effects were statistically significant (*p* < 0.01) (Table S7). The net drift was −2.07% (95% CI: −2.14%, −2.01%), indicating an overall annual decline in DALY rates after adjusting for period and cohort effects (Figure 5A). After adjusting for the period and cohort effects, the longitudinal age curve demonstrated that DALYs increased with age, peaking in the 65–69 age group, followed by a decline in older groups (Figure 5B). After controlling for age and birth cohort effects, the relative risk (RR) associated with the period effect showed an overall decrease over time (Figure 5C). For the cohort effect, after adjusting for age and period factors, RR increased in earlier birth cohorts, peaked in those born between 1920 and 1929, and then decreased (Figure 5D). Similar trends were observed for the age, period, and cohort effects on death rates (Figure S2).

### 3.5 Prediction of TBL Cancer Burden Attributable to Diet Low in Fruit

**FIGURE 6:**
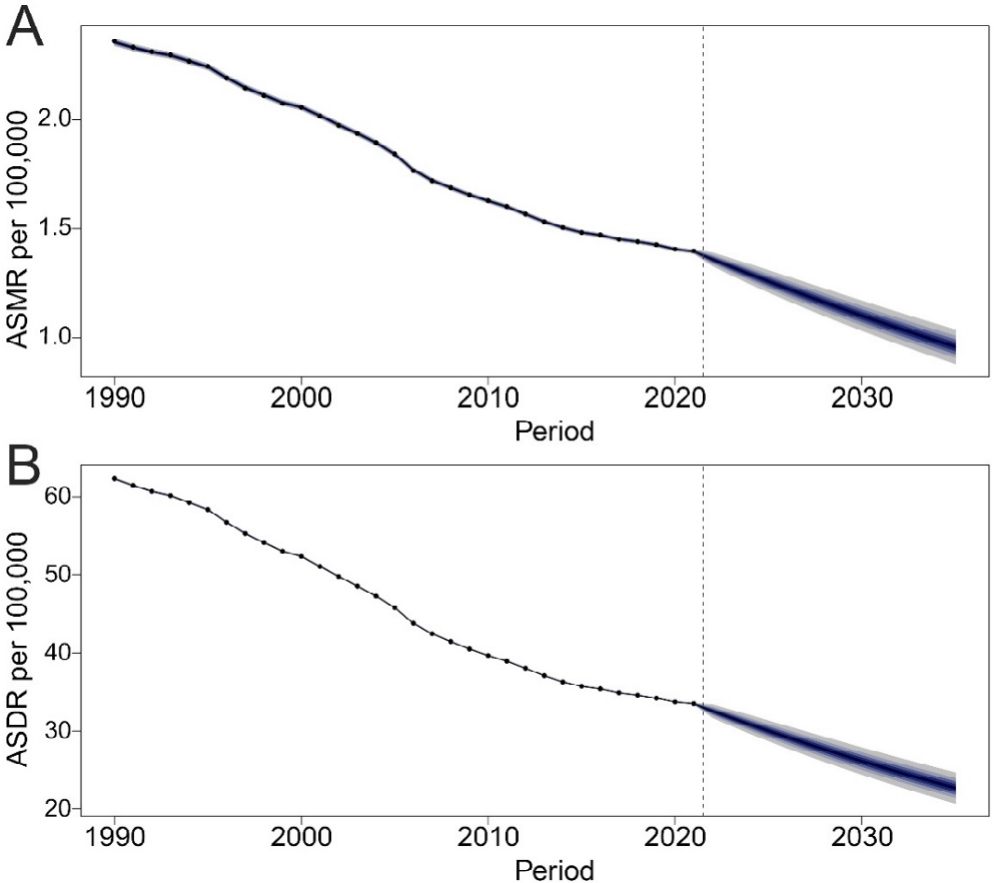
The temporal trends of ASMR and ASDR of TBL cancer attributable to diet low in fruit between 1990 and 2021, with projections up to 2035 by SDI, using BAPC prediction models. (A) ASMR globally. (B) ASDR globally. Dots represent observed values from 1990 to 2021. The solid line indicates predicted mean values, and the fan‐shaped area represents the predictive distribution between the 2.5% and 97.5% quantiles from 2022 to 2035. ASDR, age‐standardized DALYs rate; ASMR, age‐standardized mortality rate; BAPC, Bayesian age‐period‐cohort; DALYs, disability‐adjusted life years; SDI, socio‐demographic index; TBL, tracheal, bronchus, and lung.

Using the BAPC model, we projected the global trends in ASMR and ASDR of TBL cancer attributable to low fruit intake from 2022 to 2035. Both indicators are expected to continue declining over the next decade. Specifically, ASMR is projected to gradually decrease to approximately 0.96 per 100,000 by 2035 (Figure 6A), while ASDR is estimated to fall to about 22.71 per 100,000 by the same year (Figure 6B).

### 3.6 Decomposition Analysis

**FIGURE 7:**
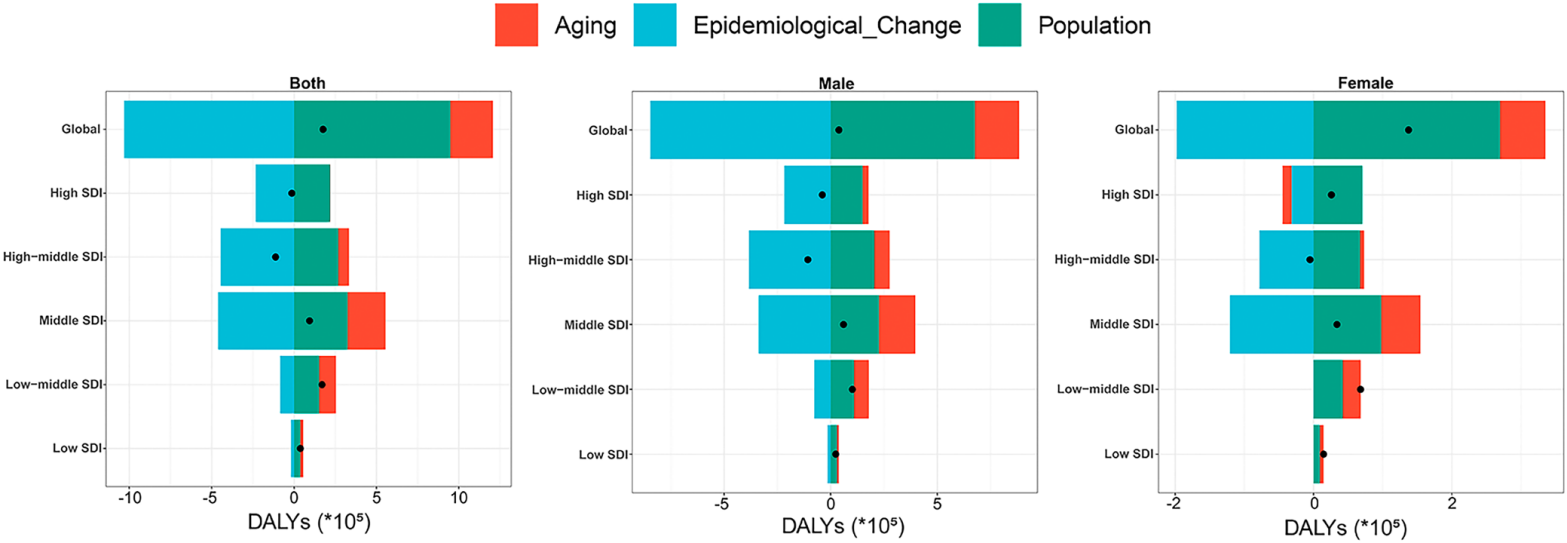
Changes in DALYs of TBL cancer attributable to diet low in fruit decomposed, decomposed by three population‐level determinants: Aging, population, and epidemiological change from 1990 to 2021, at the global level and by SDI quintiles, stratified by sex. The black dot represents the overall change contributed by all three components combined. For each component, the magnitude of a positive value indicates an increase in TBL cancer DALYs attributed to that component, while the magnitude of a negative value indicates a corresponding decrease. DALYs, disability‐adjusted life years; SDI, socio‐demographic index; TBL, tracheal, bronchus, and lung.

To quantify the relative contributions of population growth, aging, and epidemiological changes to the global and regional burden of TBL cancer attributable to diet low in fruit, we conducted a decomposition analysis. Globally, DALYs increased substantially over the past three decades, with the most marked rise observed in low‐middle SDI regions (Figure 7; Table S8). Population growth contributed the largest share to the increase (539.22%), followed by aging (146.86%). The greatest effects of both population growth (345.56%) and aging (245.03%) were found in middle SDI regions. Conversely, changes in epidemiological factors contributed to a global decrease in DALYs (−586.07%), with the strongest negative impact observed in high SDI regions (−1699.93%). These patterns were generally consistent across both sexes (Figure 7; Table S8).

### 3.7 Cross‐Country Health Inequality Analysis

**FIGURE 8:**
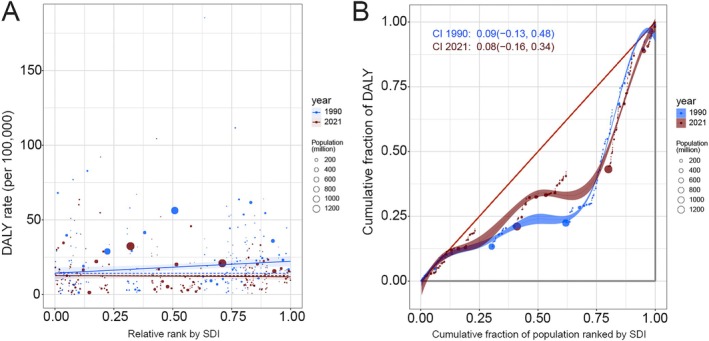
Health inequality regression curves and concentration curves for DALYs of TBL cancer attributable to diet low in fruit in 1990 and 2021. DALYs, disability‐adjusted life years; SDI, socio‐demographic index; TBL, tracheal, bronchus, and lung.

Remarkable absolute and relative SDI‐related inequalities in the burden of TBL cancer attributable to diet low in fruit were identified, with these disparities gradually decreasing over time (Figure 8). From 1990 to 2021, the absolute disparity measured by the SII decreased significantly, from 7.94 (95% CI: 2.05 to 13.84) to −0.83 (95% CI: −4.52 to 2.86) (Figure 8A). Similarly, the relative inequality indicated by the CI declined modestly, from 0.092 (95% CI: −0.125 to 0.481) in 1990 to 0.076 (95% CI: −0.164 to 0.340) in 2021 (Figure 8B).

## 4 Discussion

To our knowledge, this is the most recent study analyzing the long‐term effects of low fruit intake on mortality and DALYs attributable to TBL cancer at global, regional, and national levels. Our findings reveal that in 2021, TBL cancer attributable to low fruit intake imposed a substantial burden, with notable variations across socio‐demographic regions and between sexes. The burden was higher in males compared to females, and regions with lower SDI exhibited higher ASMR and ASDR. Temporal trend analysis indicated a global decline in the overall burden; however, certain regions continue to experience an upward trend. Population growth and aging emerged as the two major drivers of changes in DALYs. Additionally, health inequalities related to TBL cancer burden decreased across all regions during the study period. This study underscores the significant impact of TBL cancer attributable to low fruit intake on global health and offers a scientific foundation for designing targeted prevention and management strategies to mitigate this preventable burden.

Our findings linking low fruit intake to increased TBL cancer burden align with prior studies showing an inverse relationship between fruit consumption and lung cancer risk. For example, Wang et al. found a 20% risk reduction with higher intake, greater in females [6] while a Japanese analysis showed moderate consumption reduced risk in male smokers, especially where smoking prevails [7]. A dose–response meta‐analysis reported a 5% risk drop per daily serving in current smokers and 4% in former smokers [8]. These support our results and highlight fruit intake's potential to ease the global lung cancer burden. However, we must caution that the observed association between low fruit intake and TBL cancer burden does not imply causation and may be influenced by confounding factors such as smoking, air pollution, and occupational exposures—well‐established primary risk factors for TBL cancer. The Global Burden of Disease (GBD) 2021 study employs a comparative risk assessment (CRA) framework, where the population attributable fraction (PAF) for low fruit intake is derived from meta‐analyses of cohort studies that typically adjust for these confounders at the source level [16]. While this improves robustness, the PAF method inherently assumes a causal relationship, which cannot be independently verified in this study. Therefore, the attributable burden should be interpreted with caution, and further validation using individual‐level cohort data is needed. The protective effects of fruit intake against lung cancer may be attributed to several biological mechanisms. Fruits are rich in antioxidants (e.g., vitamins C, E, and beta‐carotene), phytochemicals, and dietary fiber, which help reduce oxidative stress, DNA damage, and inflammation—key processes in carcinogenesis [19, 20, 21]. Phytochemicals such as flavonoids and isothiocyanates have been shown to modulate anti‐tumor pathways, inhibit cancer cell proliferation, and induce apoptosis [22], while polyphenols and organic sulfides exert anti‐inflammatory effects that may suppress lung cancer progression [23].

The declining temporal trends in global mortality and DALY rates for TBL cancer attributable to low fruit intake reflect progress in preventive efforts, driven by improvements in socioeconomic conditions, urbanization, education, and healthcare access, which enhance nutrition and dietary awareness. Global initiatives, such as the WHO Framework Convention on Tobacco Control (FCTC), have significantly reduced smoking prevalence, a major TBL cancer risk factor [24]. Similarly, the UN Decade of Action on Nutrition (2016–2025) emphasizes healthy dietary habits, especially increased fruit and vegetable intake, to combat malnutrition and diet‐related noncommunicable diseases (NCDs) [25]. Despite these advancements, fruit intake remains below recommended levels in many regions, particularly in low and middle SDI areas [26]. This shortfall is exacerbated by the increasing reliance on processed and convenience foods that lack essential nutrients [27]. Among older adults, additional barriers such as limited access to fresh produce and physical difficulties in consuming fibrous fruits further compound the issue [28, 29]. Our AAPC analysis highlighted concerning trends in low‐middle and low SDI regions, where the TBL cancer burden attributable to low fruit intake has risen since approximately 2006, likely due to ongoing socioeconomic challenges, limited healthcare infrastructure, disparities in access to fresh fruits, and political‐economic barriers to interventions [30]. Besides dietary patterns, socioeconomic and structural factors—like limited healthcare access, air pollution, and poor occupational safety—may drive the higher burden in low‐middle SDI regions. Promoting fruit intake, a key health goal, requires improving food affordability, availability, and distribution. In low‐income areas, high costs and seasonal variability of produce hinder dietary shifts. Thus, future strategies should blend nutrition education with policies tackling structural dietary inequalities. Our BAPC predictions suggest that the global burden of TBL cancer attributable to low fruit intake will continue to decline through 2035. However, these projections are based on the assumption that current trends in dietary intake and other risk factors remain relatively stable. In reality, future changes in smoking prevalence, environmental exposures, and dietary transitions—particularly in low‐ and middle‐SDI regions—may alter the trajectory of disease burden. Sustained public health strategies, inspired by FCTC and nutrition frameworks, are essential to reduce barriers to healthy eating and mitigate this burden, particularly in vulnerable populations.

The higher TBL cancer mortality and DALY rates attributable to low fruit intake in men compared to women may reflect a mix of dietary and lifestyle factors, beyond just fruit consumption differences. Women consistently consume more fruits and vegetables than men globally [31], adopting healthier diets that likely lower their TBL cancer burden [32], while men often exhibit riskier behaviors like smoking and lower fruit intake [33]. The higher TBL cancer incidence in men, tied to greater smoking prevalence—a key risk factor—may drive this gender disparity, especially in regions like East Asia. Although the GBD 2021 adjusts for smoking in its PAF estimates [13], its aggregated data limit our ability to isolate fruit intake's specific role from confounders like smoking or alcohol use. Thus, while low fruit intake is a modifiable risk factor, its contribution to gender differences needs further study with individual‐level data. Targeted interventions—improving diets for both sexes and addressing smoking in men—remain crucial.

Population growth and aging are significant contributors to the increased burden of TBL cancer attributable to low fruit intake at global, regional, and national levels. Our decomposition analysis revealed that while epidemiological improvements, such as advancements in early detection, diagnosis, and treatment, have mitigated some of the burden, they have not fully offset the impacts of population growth and aging. This underscores the persistent and critical role of demographic factors in shaping the TBL cancer burden. Notably, aging and population growth exerted the most pronounced effects on the TBL cancer burden in middle SDI regions, reflecting distinct demographic patterns across SDI levels. The DALY burden of TBL cancer is strongly correlated with age, as aging not only increases the burden among the elderly but also shifts the age distribution of disease onset, amplifying the overall burden. Furthermore, global aging trends differ by region, affecting disease burden unevenly. According to a 2019 United Nations report on global population aging, the global elderly population (65 years and older) will exceed 1.5 billion by 2050, with Eastern and Southeastern Asia seeing the largest growth (312 million), sub‐Saharan Africa and Northern Africa/Western Asia the fastest increases (218%, 226%), and Australia/New Zealand (84%), Europe, and North America more modest growth (48%) [34]. These projections suggest that middle and low SDI regions will face a disproportionately greater impact from population aging in the coming decades, whereas the burden in high SDI regions is expected to stabilize. While aging significantly contributes to the TBL cancer burden, addressing modifiable risk factors such as fruit intake and improving preventive healthcare access remain essential for reducing future impacts, especially in rapidly aging regions [35].

Health inequalities significantly influence the burden of various diseases, including TBL cancer [36, 37]. In recent years, the interaction between dietary patterns and disease development has emerged as a critical research focus in public health [38, 39, 40]. Understanding how disparities in dietary habits and access to nutritious foods contribute to disease burden is essential for addressing inequalities and informing targeted interventions. In our study, no significant inequalities in DALY numbers were observed for TBL cancer attributable to low fruit intake. This finding may be partially explained by the fact that higher SDI countries, while exhibiting higher DALY rates, typically have smaller populations, thereby offsetting overall inequalities in DALY numbers [41]. Additionally, it is plausible that other risk factors, such as smoking and environmental exposures, play a more dominant role in influencing TBL cancer DALYs compared to dietary patterns [35].

This study has several limitations. Firstly, the GBD database aggregates TBL cancers without distinguishing histological subtypes (e.g., squamous cell carcinoma vs. adenocarcinoma), limiting subtype‐specific analysis. Secondly, the GBD assesses dietary risk factors independently, excluding interactions among dietary components (e.g., fruit and vegetable intake combined), restricting insights into overall dietary quality's role in TBL cancer risk. Thirdly, the lack of individual‐level data prevents direct adjustment for key confounders such as smoking, air pollution, and occupational exposures. While the GBD framework accounts for these through adjusted relative risks from prior studies, we could not perform stratified analyses (e.g., never‐smokers vs. smokers) to isolate their independent effects. Additionally, variability in data collection over time and across regions—particularly in low‐SDI settings where cancer registries may be sparse or inconsistent—may distort observed trends and affect the precision of EAPC estimates. Although the GBD employs Bayesian meta‐regression (DisMod‐MR 2.1) to harmonize heterogeneous data and enhance comparability, residual inconsistencies could still influence the reliability of temporal trends. Furthermore, the GBD dataset integrates multi‐source data with varying reliability, and while DisMod‐MR 2.1 mitigates some limitations, it cannot fully compensate for missing or poor‐quality data, necessitating cautious interpretation of estimates, particularly for low‐SDI regions. Finally, the PAF method assumes causality between low fruit intake and TBL cancer, which we cannot validate independently, potentially overestimating the burden if coexisting risk factors like smoking predominate. Future studies with detailed cohort data are needed to address these gaps.

## 5 Conclusion

In conclusion, our study highlights the substantial global, regional, and national burden of TBL cancer attributable to low fruit intake from 1990 to 2021. These findings emphasize the urgent need for public health initiatives to promote fruit consumption as a core component of comprehensive cancer prevention strategies. Such efforts are particularly crucial in regions with rising cancer rates, where socio‐economic and cultural barriers limit access to and consumption of fruits. Future strategies should focus on integrating dietary improvements into existing cancer prevention frameworks, addressing structural and behavioral barriers to healthy eating. By prioritizing these measures, global efforts can more effectively reduce the preventable burden of TBL cancer and improve population health outcomes.

## Author Contributions

J.‐l.L.: Conceptualization (equal); data curation (equal); formal analysis (equal); investigation (equal); methodology (lead); software (equal); funding acquisition (lead); validation (equal); visualization (equal); writing – original draft (lead). C.‐y.Z.: Data curation (equal); formal analysis (equal); investigation (equal); software (equal); validation (equal). G.‐m.P.: Formal analysis (equal); investigation (equal); software (equal); validation (equal); visualization (equal); writing – review and editing (equal). L.‐j.L.: Data curation (equal); formal analysis (equal); investigation (equal); validation (equal). J.S.: Conceptualization (equal); project administration (lead); resources (lead); supervision (lead); writing – review and editing (equal).

## Funding

The study was supported by the Medical Science and Technology Project of Zhejiang Province (2023RC289) and Shaoxing People's Hospital Cultivation Fund Project (2024PY04).

## Ethics Statement

The GBD follows the Guidelines for Accurate and Transparent Health Estimates Reporting (GATHER). Since GBD information is entirely anonymized and does not include personal data, this analysis did not require approval from a research ethics committee.

## Consent

This study did not involve direct interaction with patients or the collection of individual patient data. All data used in this study were obtained from publicly available databases, specifically the Global Burden of Disease (GBD) 2021 study. As such, no individual‐level patient consent was required.

## Conflicts of Interest

The authors declare no conflicts of interest.

## Supporting information


**Data S1:** cam471584‐sup‐0001‐Figures.docx.

**Data S2:** cam471584‐sup‐0002‐Supplementarytables.xlsx.

**Data S3:** cam471584‐sup‐0003‐supinfo.docx.

## Data Availability

The data used in this study came from a public database that everyone can access through the link provided in this article (https://vizhub.healthdata.org/gbd‐results/).
