# Supplementary material for: Global, Regional, and National Burden of Tracheal, Bronchial, and Lung Cancer Attributable to Low Fruit Intake From 1990 to 2021
Source: Cancer Med. 2026 Feb 4;15(2):e71584. doi: 10.1002/cam4.71584 (PMC12872282; doi:10.1002/cam4.71584)

**FIGURE S1.** The correlation between TBL cancer attributable to diet low in fruit in ASMR and SDI at the regional (A) and national (B) levels between 1990 and 2021. The correlation between EAPC of ASMR and ASMR in 1990 (C), and EAPC of ASMR and SDI in 2021 (D). The size of each dot corresponds to the deaths of TBL cancer attributable to diet low in fruit. The R and p values were derived from Pearson correlation analysis. ASMR, age-standardized mortality rate; TBL, tracheal, bronchus, and lung; SDI, socio-demographic index; EAPC, estimated annual percentage change.


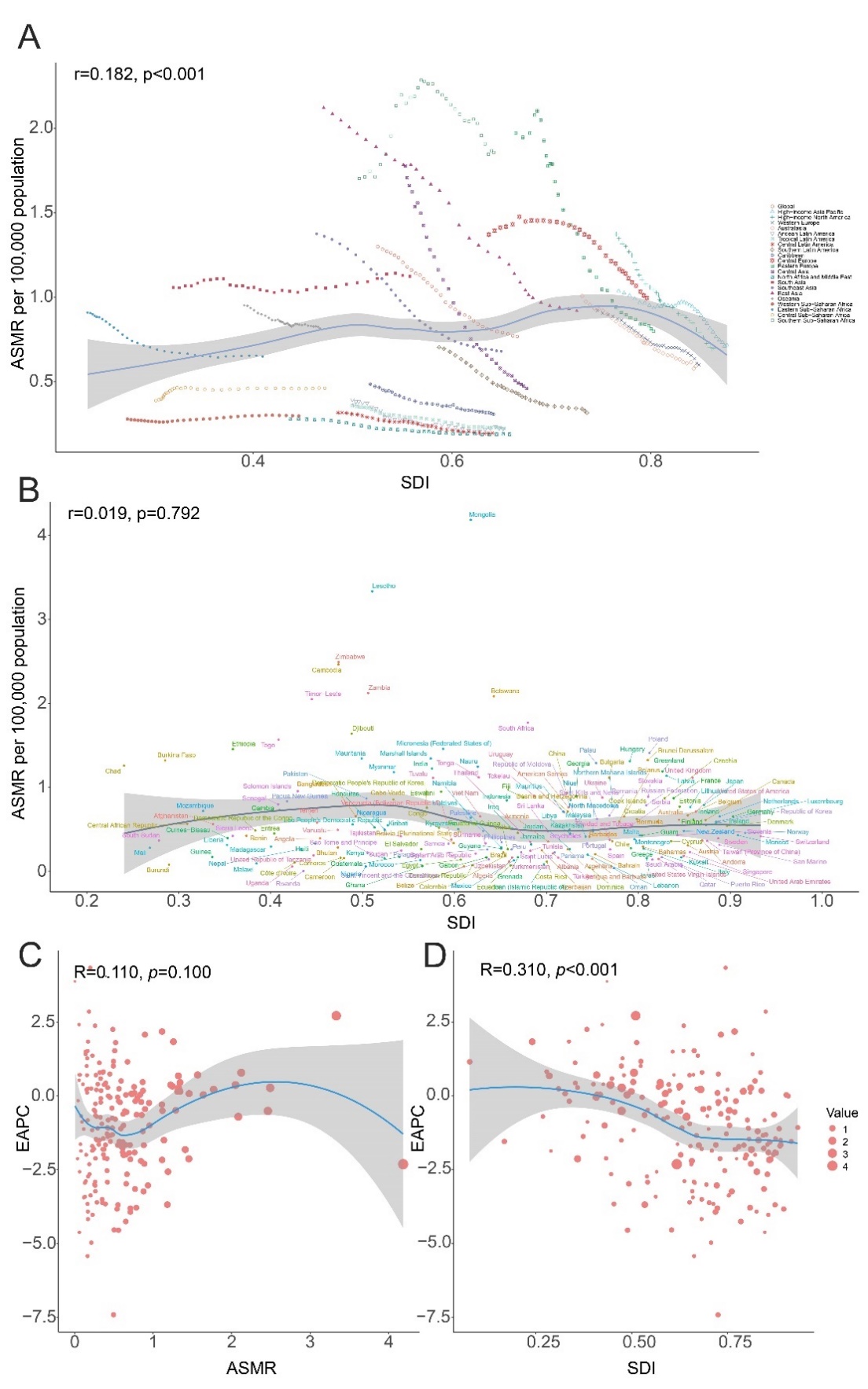


**FIGURE S2** Local drifts of TBL cancer Deaths attributable to diet low in fruit globally (A). Age-period-cohort analysis of TBL cancer Deaths attributable to diet low in fruit globally from 1990 to 2021, showing age (B), period (C), and cohort (D) effects. TBL, tracheal, bronchus, and lung.


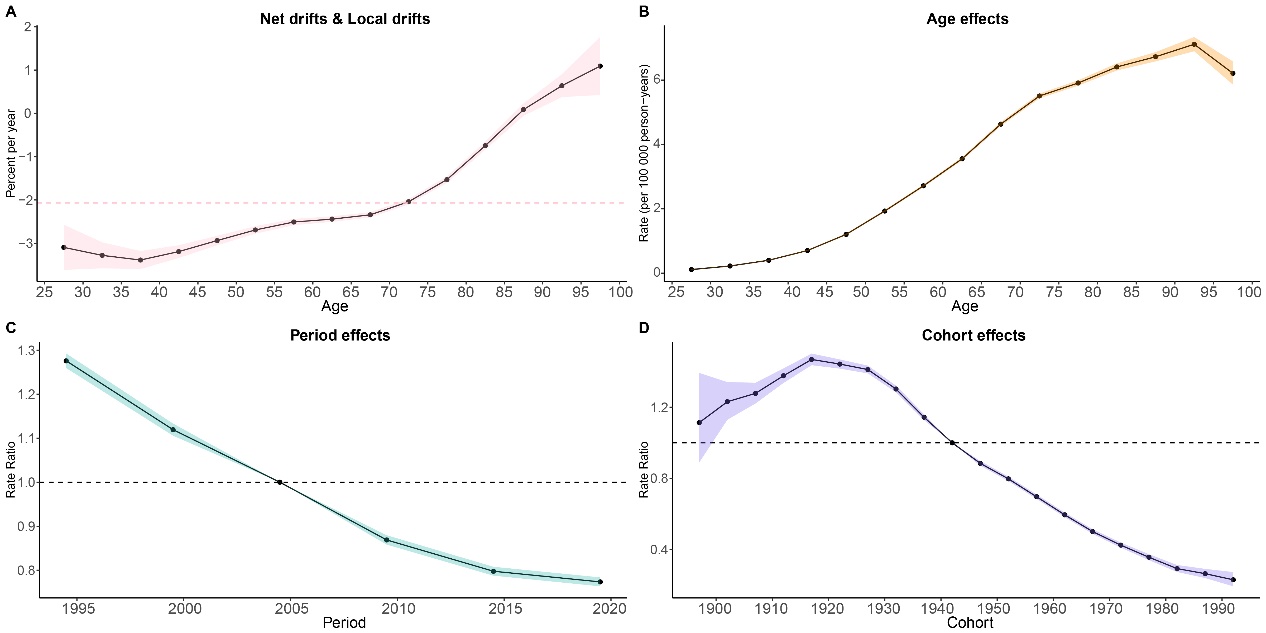

Supplement: Supplementary file 1 — Data S1: cam471584‐sup‐0001‐Figures.docx. [file CAM4-15-e71584-s003.docx]
