# Supplementary material for: Global, Regional, and National Burden of Tracheal, Bronchial, and Lung Cancer Attributable to Low Fruit Intake From 1990 to 2021
Source: Cancer Med. 2026 Feb 4;15(2):e71584. doi: 10.1002/cam4.71584 (PMC12872282; doi:10.1002/cam4.71584)
Supplement: Supplementary file 3 — Data S3: cam471584‐sup‐0003‐supinfo.docx. [file CAM4-15-e71584-s002.docx]

###############################################################################################################################################################

##agegroup

df <- fread("1521.csv")

df <- df %>%

mutate(measure_name = if_else(measure_name == "DALYs (Disability-Adjusted Life Years)", "DALYs", measure_name)) %>%

mutate(measure_name = if_else(measure_name == "YLDs (Years Lived with Disability)", "YLDs", measure_name)) %>%

filter(year > 1989)

for (i in unique(df$rei_name)) {print(i)

df1 <- df %>%

filter(rei_name == i) %>%

filter(year %in% c(1990,2021))

for (j in unique(df1$measure_name)) {print(j)

df2 <- df1 %>%

filter(measure_name==j)

for (k in unique(df2$year)) {

print(k)

dfx <- df2 %>%

filter(year == k) %>%

filter(location_name == "Global") %>%

filter(!sex_name == "Both") %>%

filter(metric_name %in% c("Rate", "Number")) %>%

filter(age_id %in% c(1, 5:20, 30, 31, 32, 235)) %>%

mutate(age_name=factor(age_name,levels=c("<5 years","5-9 years","10-14 years","15-19 years","20-24 years",

"25-29 years","30-34 years","35-39 years","40-44 years","45-49 years",

"50-54 years", "55-59 years","60-64 years" ,"65-69 years", "70-74 years",

"75-79 years", "80-84 years", "85-89 years", "90-94 years", "95+ years")))

dfx=dfx

labx="Number of cases"

color_scheme = 2

#agex=c(1,5:20,30,31,32,235)

# Define the color palettes as dataframes

color_palettes <- data.frame(

palette = c(1, 2, 3, 4, 5),

color1 = c("#EC7064"),

color2 = c("#2372A3"),

legend_fill2 = rep("Male(number and 95%UI)",5),

legend_fill1 = rep("Female(number and 95%UI)",5),

legend_color2 = rep("Male(rate and 95%UI)",5),

legend_color1 = rep("Female(rate and 95%UI)",5)

)

dfx=dfx %>% mutate(age_name = if_else(age_name %in% c("80-84", "85-89", "90-94"),

paste(age_name, "years"),

age_name))

dfage=dfage %>% mutate(age_name = if_else(age_name %in% c("80-84", "85-89", "90-94"),

paste(age_name, "years"),

age_name))

library(ggplot2)

library(dplyr)

# Check the number of unique location_id and cause_id

if (length(unique(dfx$age_id)) < 2 || length(unique(dfx$year)) > 1) {

p= ggplot() +

geom_text(aes(x = 0.5, y = 0.5, label = "Oops, your data is not suitable for this plot"), size = 12) +

theme_void()

return(p)

}

dfid=dfx %>% select(age_id,age_name) %>% distinct(age_id,.keep_all = T) %>%

left_join(.,dfage) %>% arrange(id)

dfx=left_join(dfx,dfid) %>% filter(!is.na(id))

dfx$age_name <- factor(dfx$age_name, levels = unique(dfid$age_name[order(dfid$id)]))

df1=dfx #%>% filter(age_id %in% agex) %>% mutate(age_name=factor(age_name,levels=x8$age_name))

# Load the ggplot2 package

# First, let's prepare the data by dividing it into two separate data frames: one for Number and one for Rate.

df1_number <- df1 %>% filter(metric_name == "Number") #%>% left_join(.,x8)

df1_rate <- df1 %>% filter(metric_name == "Rate") #%>% left_join(.,x8)

# We will normalize the 'val' for Rate to the same scale as Number for visual purposes.

max_number <- max(df1_number$val)

max_rate <- max(df1_rate$val)

scaling_factor <- max_number / max_rate

# Start the plot

x1=color_palettes %>% filter(palette==color_scheme)

sexx=unique(dfx$sex_name)

# Create a function to select the appropriate color palette

if ("Male" %in% sexx && "Female" %in% sexx) {

x1=x1

} else if (sexx == "Male") {

x1$legend_fill2 <- "Male(number and 95%UI)"

x1$legend_fill1 <- "Male(number and 95%UI)"

x1$legend_color2 <- "Male(rate and 95%UI)"

x1$legend_color1 <- "Male(number and 95%UI)"

} else {

x1$legend_fill2 <- "Female(number and 95%UI)"

x1$legend_fill1 <- "Female(number and 95%UI)"

x1$legend_color2 <- "Female(rate and 95%UI)"

x1$legend_color1 <- "Female(number and 95%UI)"

}

xa=ggplot() +

geom_bar(data = df1_number, aes(x = age_name, y = val, fill = sex_name),

stat = "identity", position = position_dodge(), color = "black") +

geom_errorbar(data = df1_number, aes(x = age_name, ymin = lower, ymax = upper, group = sex_name),

position = position_dodge(0.9), width = 0.25) +

geom_line(data = df1_rate, aes(x = age_name, y = val * scaling_factor, group = sex_name, color = sex_name),

position = position_dodge(0.9)) +

geom_ribbon(data = df1_rate, aes(x = age_name, ymin = lower * scaling_factor, ymax = upper * scaling_factor, group = sex_name, fill = sex_name),

alpha = 0.5, position = position_dodge(width = 0.9)) + # Ensure ribbons are displayed for rates

labs(x="",y = labx) +

# Set primary and secondary y-axes

scale_y_continuous(labels = label_number(unit = "K"),sec.axis = sec_axis(~./scaling_factor, name = "Rate per 100,000 population")) +

#ggsci::scale_fill_aaas(name = " ")+

scale_fill_manual(values = c(x1$color1,x1$color2),labels =c(x1$legend_fill1,x1$legend_fill2),name=" ") +

scale_color_manual(values = c(x1$color1,x1$color2),labels = c(x1$legend_color1,x1$legend_color2),name=" ") +

theme_classic() +

theme(legend.position = "top",

# 调整字体大小和加粗

axis.text.x = element_text(angle = 65, hjust = 1, size = 12, face = "bold"), # X轴标签

axis.text.y = element_text(size = 12, face = "bold"), # Y轴标签

axis.title.x = element_text(size = 14, face = "bold"), # X轴标题

axis.title.y = element_text(size = 14, face = "bold"), # Y轴标题

# 调整图例的字体大小和加粗

legend.text = element_text(size = 12, face = "bold"), # 图例文本

legend.title = element_text(size = 14, face = "bold") # 图例标题

)

# 打印生成的图表

print(xa)

# 保存图表

ggsave(paste0("agegroup-",i,"-",j,"-",k,".pdf"), width = 14, height = 10)

# 保存 CSV 数据

write.csv(dfx, paste0("agegroup-",i,"-",j,"-",k,".csv"))

} } }

###############################################################################################################################################################

###21SDI

df <- read.csv("1521.csv") %>%

mutate(measure_name = if_else(measure_name == "DALYs (Disability-Adjusted Life Years)", "DALYs", measure_name)) %>%

mutate(measure_name = if_else(measure_name == "YLDs (Years Lived with Disability)", "YLDs", measure_name)) %>%

filter(year>1989)

q <- "All causes"

df1 <- df %>%

filter(cause_name == q)

# 遍历 `measure_name`，生成子图

for (i in unique(df1$measure_name)[c(1)]) {

print(i)

dfx <- df1 %>%

filter(measure_name == i) %>%

filter(location_name %in% loc21) %>%

filter(sex_name == "Both") %>%

filter(age_id == "27") %>%

filter(metric_name == "Rate")

dfq <- dfx %>% select("measure_name","location_name","sex_name","age_name", "cause_name",

"rei_name", "metric_name", "year", "val" )

dfqq <- left_join(dfq,SDI2019) %>% distinct(sdi, .keep_all = TRUE)

write.csv(dfqq,paste0("21SDI-",i,"-.csv"))

dfx = dfx

labx = "xxxx"

# 定义颜色

colors <- c(

pal_npg("nrc", alpha = 0.7)(9),

pal_aaas("default", alpha = 0.7)(9),

pal_nejm("default", alpha = 0.7)(8),

pal_jama("default", alpha = 0.7)(7)

)

# 数据处理

DALY_2017 = dfx %>% select(location_id, val, year)

df11 = left_join(DALY_2017, SDI2019)

df3 = df11 %>%

filter(sdi > 0.2) %>%

mutate(location_id = factor(location_id, levels = c(

1, 5, 9, 21, 32, 42, 56, 65, 70, 73, 96, 100, 104, 120, 124, 134, 138, 159,

167, 174, 192, 199

)))

# 计算 R 值和 P 值

cor_result <- cor.test(df3$sdi, df3$val)

r_value <- cor_result$estimate # 相关系数 R

p_value <- cor_result$p.value # P 值

# 格式化 P 值显示

p_label <- ifelse(

is.na(p_value), "P = NA",

ifelse(p_value < 0.001, "P < 0.001",

ifelse(p_value < 0.01, "P < 0.01",

ifelse(p_value < 0.05, "P < 0.05",

paste0("P = ", round(p_value, 3))))))

# 格式化 R 值显示

r_label <- paste0("R = ", round(r_value, 3))

# 绘图

p = ggplot(data = df3, aes(x = sdi, y = val)) +

geom_point(aes(shape = as.factor(location_id), color = as.factor(location_id)), size = 2) +

scale_shape_manual(

values = c(1:22),

breaks = c(

1, 5, 9, 21, 32, 42, 56, 65, 70, 73, 96, 100, 104, 120, 124, 134, 138, 159,

167, 174, 192, 199

),

labels = c(

"Global", "High-income Asia Pacific", "High-income North America", "Western Europe",

"Australasia", "Andean Latin America", "Tropical Latin America", "Central Latin America",

"Southern Latin America", "Caribbean", "Central Europe", "Eastern Europe", "Central Asia",

"North Africa and Middle East", "South Asia", "Southeast Asia", "East Asia", "Oceania",

"Western Sub-Saharan Africa", "Eastern Sub-Saharan Africa", "Central Sub-Saharan Africa",

"Southern Sub-Saharan Africa"

)

) +

scale_color_manual(

values = colors[1:22],

breaks = c(

1, 5, 9, 21, 32, 42, 56, 65, 70, 73, 96, 100, 104, 120, 124, 134, 138, 159,

167, 174, 192, 199

),

labels = c(

"Global", "High-income Asia Pacific", "High-income North America", "Western Europe",

"Australasia", "Andean Latin America", "Tropical Latin America", "Central Latin America",

"Southern Latin America", "Caribbean", "Central Europe", "Eastern Europe", "Central Asia",

"North Africa and Middle East", "South Asia", "Southeast Asia", "East Asia", "Oceania",

"Western Sub-Saharan Africa", "Eastern Sub-Saharan Africa", "Central Sub-Saharan Africa",

"Southern Sub-Saharan Africa"

)

) +

geom_smooth(span = 0.8, se = FALSE, color = "black") +

labs(

shape = "",

color = "",

x = paste0("SDI"),

y = paste0(labx, " Rate per 100,000 population")

) +

theme_bw() +

theme(

legend.key.size = unit(0.03, "line"),

panel.border = element_blank(),

panel.grid.major = element_blank(),

panel.grid.minor = element_blank(),

legend.background = element_blank(),

legend.position = "right",

# 加粗标题和标签字体

legend.title = element_text(size = 20, face = "bold"),

axis.title = element_text(size = 24, face = "bold"),

axis.text = element_text(size = 20, face = "bold"),

legend.text = element_text(size = 20, face = "bold"),

# 图表标题

plot.title = element_text(size = 26, face = "bold", hjust = 0.5),

plot.subtitle = element_text(size = 20, face = "bold"),

# 坐标轴线

axis.line = element_line(colour = "black")

) +

guides(color = guide_legend(ncol = 1),

shape = guide_legend(ncol = 1))+

# 左上角添加 R 和 P 值

annotate("text", x = min(df3$sdi), y = max(df3$val),

label = paste0(r_label, "\n", p_label),

hjust = -0.5, vjust = 1, size = 6, fontface = "bold")

print(p)

ggsave(paste0("21SDI-",i,"-.pdf"), plot = p, width = 12, height = 8)

}

###############################################################################################################################################################

###204sdi

df=fread("204.csv",header = T) %>%

mutate(measure_name = if_else(measure_name == "DALYs (Disability-Adjusted Life Years)", "DALYs", measure_name)) %>%

mutate(measure_name = if_else(measure_name == "YLDs (Years Lived with Disability)", "YLDs", measure_name)) %>%

filter(year>1989)

i <- "YLDs"

df1 <- df %>%

filter(measure_name ==i) %>%

filter(location_id %in% name_204) %>%

filter(!metric_name == "Percent") %>%

filter(year %in% c(2021)) %>%

filter(age_id == 27) %>%

filter(metric_name == "Rate") %>%

filter(sex_name=="Both")

dfx = dfx

labx = "xxx"

# 如果数据为空时显示提示

if (nrow(dfx) == 0) {

p1 = ggplot() +

geom_text(aes(x = 0.5, y = 0.5, label = "Oops, your data is not suitable for this plot"),

size = 12, fontface = "bold") + # 加粗字体

theme_void()

spearx = "No data"

return(list(p = p1, spearx = spearx))

}

# 数据处理

GBD1990_2019 = dfx %>%

select(location_id, val, year)

SDI2019 = SDI2019 %>%

left_join(., GBD1990_2019) %>%

filter(!is.na(val))

# 计算 Spearman 的相关系数

spearman_cor <- cor.test(SDI2019$sdi, SDI2019$val, method = "spearman")

r <- spearman_cor$estimate

p <- spearman_cor$p.value

# 动态生成 P 值标签

p_label <- ifelse(

is.na(p), "P = NA",

ifelse(p < 0.001, "P < 0.001",

ifelse(p < 0.01, "P < 0.01",

ifelse(p < 0.05, "P < 0.05",

paste0("P = ", round(p, 3))))))

# 动态生成 R 值和 P 值的文本

annotation_text <- sprintf("R = %.2f\n%s", r, p_label)

# 绘图

p1 = ggplot(SDI2019, aes(x = sdi, y = val)) +

geom_point(aes(col = location_name), size = 1.2) +

geom_smooth(method = "loess", se = TRUE, color = "#708090") +

geom_text_repel(aes(label = location_name, col = location_name),

size = 4, max.overlaps = 60, segment.size = 0.2) + # 加粗字体

labs(

x = paste0("SDI (", 2019, ")"),

y = paste0(labx, "\n per 100,000 population")

) +

theme_bw() +

theme(

legend.position = "none",

panel.border = element_blank(),

panel.grid.major = element_blank(),

panel.grid.minor = element_blank(),

axis.line = element_line(colour = "black"),

# 统一放大并加粗字体

axis.title = element_text(size = 24, face = "bold"), # 坐标轴标题

axis.text = element_text(size = 20, face = "bold"), # 坐标轴刻度标签

plot.title = element_text(size = 26, face = "bold", hjust = 0.5), # 图形标题

plot.subtitle = element_text(size = 22, face = "bold"), # 图形副标题

legend.title = element_text(size = 20, face = "bold"), # 图例标题

legend.text = element_text(size = 18, face = "bold") # 图例文本

) +

scale_x_continuous(name = "SDI ", limits = c(0.2, 1.0), breaks = seq(0, 1, 0.1)) +

annotate("text", x = 0.25, y = max(SDI2019$val, na.rm = TRUE),

label = annotation_text, hjust = 0, vjust = 1, size = 8, color = "black", fontface = "bold") # 加粗注释字体

# 返回图形和相关系数

spearx <- sprintf("r=%.4f, p=%.3e", r, p)

write.csv(df2,paste0("204SDI-",i,"-.csv"))

ggsave(paste0("204SDI-",i,"-.pdf"), plot = p1, width = 16, height = 12)

df <- read.csv("204.csv", header = TRUE) %>%

filter(metric_name=="Rate") %>%

filter(age_name=="Age-standardized") %>%

filter(location_id %in% name_204) %>%

filter(sex_name=="Both")

# 定义计算 EAPC 的函数

dfeapc <- function(data) {

# 确保年和 val 列不包含 NA，并按年份排序

data_clean <- data %>%

filter(!is.na(year) & !is.na(val)) %>%

arrange(year)

# 拟合线性回归模型：log(val) ~ year

model <- lm(log(val) ~ year, data = data_clean)

# 提取回归系数 (year 的系数即为 β 值)

beta <- coef(model)["year"]

# 计算 EAPC

eapc <- (exp(beta) - 1) * 100

return(eapc)

}

# 计算所有组合的 EAPC

# 可以按 location_name, sex_name, age_name, cause_name 等分组计算 EAPC

eapc_results <- df %>%

group_by(location_id,location_name, sex_name, age_name, cause_name,measure_name) %>%

summarise(val = dfeapc(cur_data()))

dfx <- eapc_results %>% filter(measure_name=="Deaths")

dfq <- SDI2019

# Ensure you have the 'readxl' package installed

install.packages("readxl")

library(readxl)

# Load the Excel file into R

file_path <- SDI2019

dfq <- read_excel(file_path)

dfa <- dfq %>% rename(location_name = country,SDI = value) %>%

select(location_name,SDI)

# View the first few rows to check if the data has been loaded correctly

head(dfa)

# 合并数据框

merged_df <- dfa %>%

left_join(dfx, by = "location_name")

# 查看合并后的数据

head(merged_df)

# 计算Spearman相关系数和P值

spearman_test <- cor.test(merged_df$SDI, merged_df$val, method = "spearman")

# 提取Spearman ρ值和P值

rho_value <- round(spearman_test$estimate, 2)

p_value <- spearman_test$p.value

# 根据p值范围设置显示内容

p_value_label <- ifelse(p_value < 0.001, "P < 0.01",

ifelse(p_value < 0.05, "P < 0.05", "P > 0.05"))

# 绘制图形，将图例标签设置为空白，并调整点的大小范围

p1 <- ggplot(merged_df, aes(x = SDI, y = val, size = abs(val))) + # 使用abs(val)确保大小为正

geom_point(color = "#F08080", alpha = 0.7) + # 设置散点颜色和透明度

geom_smooth(method = "loess", color = "black", se = TRUE, show.legend = FALSE) + # 使用loess进行拟合

labs(title = "Age-standardized DALYs rate",

subtitle = paste0("ρ = ", rho_value, ", ", p_value_label), # 将ρ值和P值标签添加到副标题

x = "SDI",

y = "EAPC",

size = "") + # 将图例文字设置为空

scale_size_continuous(range = c(1, 8), guide = guide_legend()) + # 调整点的大小范围并显示图例

theme_classic(base_size = 14) + # 设置主题样式

theme(plot.subtitle = element_text(size = 16, hjust = 1, color = "black"))

# 打印图形

print(p1)

ggsave(paste0("eapc-SDI-", "Deaths", ".pdf"), plot = p1, width = 12, height = 8)

write.csv(merged_df, paste0("eapc-SDI-", "Deaths", ".csv"))

df <- read.csv("risk-NAFLD-204.csv", header = TRUE) %>%

filter(metric_name=="Rate") %>%

filter(age_name=="Age-standardized") %>%

filter(location_id %in% name_204) %>%

filter(sex_name=="Both")

# 定义计算 EAPC 的函数

dfeapc <- function(data) {

# 确保年和 val 列不包含 NA，并按年份排序

data_clean <- data %>%

filter(!is.na(year) & !is.na(val)) %>%

arrange(year)

# 拟合线性回归模型：log(val) ~ year

model <- lm(log(val) ~ year, data = data_clean)

# 提取回归系数 (year 的系数即为 β 值)

beta <- coef(model)["year"]

# 计算 EAPC

eapc <- (exp(beta) - 1) * 100

return(eapc)

}

# 计算所有组合的 EAPC

# 可以按 location_name, sex_name, age_name, cause_name 等分组计算 EAPC

eapc_results <- df %>%

group_by(location_id,location_name, sex_name, age_name, cause_name,measure_name) %>%

summarise(val = dfeapc(cur_data()))

dfx <- eapc_results %>% filter(measure_name=="DALYs (Disability-Adjusted Life Years)")

dfq <- "/Users/mac/Desktop/GBD最新版本/hdr-data.xlsx"

# Ensure you have the 'readxl' package installed

install.packages("readxl")

library(readxl)

# Load the Excel file into R

file_path <- "/Users/mac/Desktop/GBD最新版本/hdr-data.xlsx"

dfq <- read_excel(file_path)

dfa <- dfq %>% rename(location_name = country,SDI = value) %>%

select(location_name,SDI)

# View the first few rows to check if the data has been loaded correctly

head(dfa)

# 合并数据框

merged_df <- dfa %>%

left_join(dfx, by = "location_name")

# 查看合并后的数据

head(merged_df)

# 安装并加载所需的包

# 计算Spearman相关系数和P值

spearman_test <- cor.test(merged_df$SDI, merged_df$val, method = "spearman")

# 提取Spearman ρ值和P值

rho_value <- round(spearman_test$estimate, 2)

p_value <- spearman_test$p.value

# 根据p值范围设置显示内容

p_value_label <- ifelse(p_value < 0.001, "P < 0.01",

ifelse(p_value < 0.05, "P < 0.05", "P > 0.05"))

# 绘制图形，将图例标签设置为空白，并调整点的大小范围

p1 <- ggplot(merged_df, aes(x = SDI, y = val, size = abs(val))) + # 使用abs(val)确保大小为正

geom_point(color = "#F08080", alpha = 0.7) + # 设置散点颜色和透明度

geom_smooth(method = "loess", color = "black", se = TRUE, show.legend = FALSE) + # 使用loess进行拟合

labs(title = "Age-standardized DALYs rate",

subtitle = paste0("ρ = ", rho_value, ", ", p_value_label), # 将ρ值和P值标签添加到副标题

x = "SDI",

y = "EAPC",

size = "") + # 将图例文字设置为空

scale_size_continuous(range = c(1, 8), guide = guide_legend()) + # 调整点的大小范围并显示图例

theme_classic(base_size = 14) + # 设置主题样式

theme(plot.subtitle = element_text(size = 16, hjust = 1, color = "black"))

# 打印图形

print(p1)

ggsave(paste0("eapc-SDI-", "Dalys", ".pdf"), plot = p1, width = 12, height = 8)

write.csv(merged_df, paste0("eapc-SDI-", "Dalys", ".csv"))

df <- read.csv("risk-NAFLD-204.csv", header = TRUE) %>%

filter(metric_name=="Rate") %>%

filter(age_name=="Age-standardized") %>%

filter(location_id %in% name_204) %>%

filter(sex_name=="Both")

# 定义计算 EAPC 的函数

dfeapc <- function(data) {

# 确保年和 val 列不包含 NA，并按年份排序

data_clean <- data %>%

filter(!is.na(year) & !is.na(val)) %>%

arrange(year)

# 拟合线性回归模型：log(val) ~ year

model <- lm(log(val) ~ year, data = data_clean)

# 提取回归系数 (year 的系数即为 β 值)

beta <- coef(model)["year"]

# 计算 EAPC

eapc <- (exp(beta) - 1) * 100

return(eapc)

}

# 计算所有组合的 EAPC

# 可以按 location_name, sex_name, age_name, cause_name 等分组计算 EAPC

eapc_results <- df %>%

group_by(location_id,location_name, sex_name, age_name, cause_name,measure_name) %>%

summarise(val = dfeapc(cur_data()))

dfx <- eapc_results %>% filter(measure_name=="DALYs (Disability-Adjusted Life Years)")

dfq <- read.csv("risk-NAFLD-204.csv", header = TRUE) %>%

filter(metric_name=="Rate") %>%

filter(age_name=="Age-standardized") %>%

filter(location_id %in% name_204) %>%

filter(sex_name=="Both") %>%

filter(year==2021)

# Ensure you have the 'readxl' package installed

# Load the Excel file into R

dfa <- dfq %>% rename(ASR = val) %>% filter(measure_name=="DALYs (Disability-Adjusted Life Years)") %>%

select(location_name,ASR)

# View the first few rows to check if the data has been loaded correctly

head(dfa)

# 合并数据框

merged_df <- dfa %>%

left_join(dfx, by = "location_name")

# 查看合并后的数据

head(merged_df)

# 计算Spearman相关系数和P值

spearman_test <- cor.test(merged_df$ASR, merged_df$val, method = "spearman")

# 提取Spearman ρ值和P值

rho_value <- round(spearman_test$estimate, 2)

p_value <- spearman_test$p.value

# 根据p值范围设置显示内容

p_value_label <- ifelse(p_value < 0.001, "P < 0.01",

ifelse(p_value < 0.05, "P < 0.05", "P > 0.05"))

# 绘制图形，将图例标签设置为空白，并调整点的大小范围

# 绘制图形，使用loess进行非线性拟合

p1 <- ggplot(merged_df, aes(x = ASR, y = val, size = abs(val))) + # 使用abs(val)确保大小为正

geom_point(color = "#F08080", alpha = 0.7) + # 设置散点颜色和透明度

geom_smooth(method = "loess", color = "black", se = TRUE, show.legend = FALSE) + # 使用loess进行拟合

labs(title = "Age-standardized DALYs rate",

subtitle = paste0("ρ = ", rho_value, ", ", p_value_label), # 将ρ值和P值标签添加到副标题

x = "ASR",

y = "EAPC",

size = "") + # 将图例文字设置为空

scale_size_continuous(range = c(1, 8), guide = guide_legend()) + # 调整点的大小范围并显示图例

theme_classic(base_size = 14) + # 设置主题样式

theme(plot.subtitle = element_text(size = 16, hjust = 1, color = "black"))

# 打印图形

print(p1)

ggsave(paste0("eapc-", "Dalys", ".pdf"), plot = p1, width = 12, height = 8)

write.csv(merged_df, paste0("eapc-", "Dalys", ".csv"))

###############################################################################################################################################################

##map

# nation_wordplot ---------------------------------------------------------

fxnation <- read.csv("204.csv")

str(fxnation)

library(tidyverse)

library(sf)

library(ggplot2)

location <- read.csv("location.csv")

map <- read_sf("世界国家.shp")

map <- st_set_crs(map,4326)

GBD <- changeold_nem

GBD <- left_join(GBD,location,by="location")

GBD <- left_join(map,GBD,by=c("FENAME"="location2"))

GBDna <- na.omit(GBD)

#数据准备

GBDincidence <- GBDna |> filter(measure=="Incidence")

GBDDeaths <- GBDna |> filter(measure=="Deaths")

GBDdaly <- GBDna |> filter(measure=="DALYs (Disability-Adjusted Life Years)")

ggplot(data=GBDna)+geom_sf(aes(group=NAME,fill=changerate),color='black',size = 0.5) +

theme_void()++scale_fill_bmj()+scale_fill_manual(values = 1:8)

# futu --------------------------------------------------------------------

#附图制作

worldData <- map_data('world')

small_map_data <- changeold_nem |> filter(measure=="Incidence")

small_map_data$location[small_map_data$location == 'United States of America'] = 'USA'

small_map_data$location[small_map_data$location == 'Russian Federation'] = 'Russia'

small_map_data$location[small_map_data$location == 'United Kingdom'] = 'UK'

small_map_data$location[small_map_data$location == 'Congo'] = 'Republic of Congo'

small_map_data$location[small_map_data$location == "Iran (Islamic Republic of)"] = 'Iran'

small_map_data$location[small_map_data$location == "Democratic People's Republic of Korea"]= 'North Korea'

small_map_data$location[small_map_data$location == "Taiwan (Province of China)"] = 'Taiwan'

small_map_data$location[small_map_data$location == "Republic of Korea"] = 'South Korea'

small_map_data$location[small_map_data$location == "United Republic of Tanzania"] ='Tanzania'

small_map_data$location[small_map_data$location == "Bolivia (Plurinational State of)"] ='Bolivia'

small_map_data$location[small_map_data$location == "Venezuela (Bolivarian Republic of)"] ='Venezuela'

small_map_data$location[small_map_data$location == "Czechia"] = 'Czech Republic'

small_map_data$location[small_map_data$location == "Republic of Moldova"] = 'Moldova'

small_map_data$location[small_map_data$location == "Viet Nam"] = 'Vietnam'

small_map_data$location[small_map_data$location == "Lao People's Democratic Republic"] ='Laos'

small_map_data$location[small_map_data$location == "Syrian Arab Republic"] = 'Syria'

small_map_data$location[small_map_data$location == "North Macedonia"] = 'Macedonia'

small_map_data$location[small_map_data$location == "Micronesia (Federated States of)"] ='Micronesia'

small_map_data$location[small_map_data$location == "Macedonia"] = 'North Macedonia'

small_map_data$location[small_map_data$location == "Trinidad and Tobago"] = 'Trinidad'

a <- small_map_data[small_map_data$location == "Trinidad",]

a$location <- 'Tobago'

small_map_data <- rbind(small_map_data,a)

small_map_data$location[small_map_data$location == "Cabo Verde"] = 'Cape Verde'

small_map_data$location[small_map_data$location == "United States Virgin Islands"] ='Virgin Islands'

small_map_data$location[small_map_data$location == "Antigua and Barbuda"] = 'Antigu'

a <- small_map_data[small_map_data$location == "Antigu",]

a$location <- 'Barbuda'

small_map_data <- rbind(small_map_data,a)

small_map_data$location[small_map_data$location == "Saint Kitts and Nevis"] = 'Saint Kitts'

a <- small_map_data[small_map_data$location == "Saint Kitts",]

a$location <- 'Nevis'

small_map_data <- rbind(small_map_data,a)

small_map_data$location[small_map_data$location == "Côte d'Ivoire"] = 'Ivory Coast'

small_map_data$location[small_map_data$location == "Saint Vincent and the Grenadines"] =

'Saint Vincent'

a <- small_map_data[small_map_data$location == "Saint Vincent",]

a$location <- 'Grenadines'

small_map_data <- rbind(small_map_data,a)

small_map_data$location[small_map_data$location == "Eswatini"] = 'Swaziland'

small_map_data$location[small_map_data$location == "Brunei Darussalam"] = 'Brunei'

small_map_data <- full_join(worldData,small_map_data,by = c('region'='location')) %>%

filter(val != "NA")

fig <- ggplot(data=small_map_data)+geom_polygon(aes(x = long, y = lat,group = group,fill=changerate),

colour="black",size=0.5)+theme_bw()+

theme(axis.title.x = element_blank(),

axis.text.x = element_blank(),

axis.ticks.x = element_blank(),axis.text.y = element_blank(),

axis.ticks.y = element_blank(),axis.title.y = element_blank())+

labs(title ="A Change in incidence cases" )

fxp2<- fig+ labs(x=" ",y="",title="Caribbean and central America")+

coord_cartesian(xlim = c(-92,-60),ylim = c(5,27))+theme(panel.border = element_rect(color='black',fill=NA,size = 0.5))+

theme(legend.position = "none")

fxp3 <- fig+ labs(x=" ",y="",title="Persian Gulf")+

coord_cartesian(xlim = c(45,55),ylim = c(19,31))+theme(panel.border = element_rect(color='black',fill=NA,size = 0.5))+

theme(legend.position = "none")

fxp4 <- fig+ labs(x=" ",y="",title="Balkan Peninsula")+

coord_cartesian(xlim = c(12,32),ylim = c(35,53))+theme(panel.border = element_rect(color='black',fill=NA,size = 0.5))+

theme(legend.position = "none")

fxp5 <- fig+ labs(x=" ",y="",title="Sotheast Asia")+

coord_cartesian(xlim = c(98,123),ylim = c(-10,8))+theme(panel.border = element_rect(color='black',fill=NA,size = 0.5))+

theme(legend.position = "none")

fxp6 <- fig+ labs(x=" ",y="",title="West Africa") +

coord_cartesian(xlim = c(-17,-7),ylim = c(7,20))+theme(panel.border = element_rect(color='black',fill=NA,size = 0.5))+

theme(legend.position = "none")

fxp7 <- fig+ labs(x=" ",y="",title="Eastern \nMediterranean")+

coord_cartesian(xlim = c(32,37),ylim = c(29,35))+theme(panel.border = element_rect(color='black',fill=NA,size = 0.5))+

theme(legend.position = "none")

fxp8 <- fig+ labs(x=" ",y="",title="Northern Europe",size=4) +

coord_cartesian(xlim = c(5,25),ylim = c(48,60))+theme(panel.border = element_rect(color='black',fill=NA,size = 0.5))+

theme(legend.position = "none")

A=(fxp6|fxp7)/fxp8

A

plot<- fig +

(fxp2+fxp3+fxp4+fxp5+A+plot_layout(ncol = 5,widths=c(1.5,1,1.1,1.2,1)))+

plot_layout(ncol = 1,heights = c(9, 3))

plot

ggsave("changenation_incidence.jpeg",width=16,height=9,dpi=450)

#nation2

worldData <- map_data('world')

small_map_data <- changeold_nem |> filter(measure=="Deaths")

small_map_data$location[small_map_data$location == 'United States of America'] = 'USA'

small_map_data$location[small_map_data$location == 'Russian Federation'] = 'Russia'

small_map_data$location[small_map_data$location == 'United Kingdom'] = 'UK'

small_map_data$location[small_map_data$location == 'Congo'] = 'Republic of Congo'

small_map_data$location[small_map_data$location == "Iran (Islamic Republic of)"] = 'Iran'

small_map_data$location[small_map_data$location == "Democratic People's Republic of Korea"]= 'North Korea'

small_map_data$location[small_map_data$location == "Taiwan (Province of China)"] = 'Taiwan'

small_map_data$location[small_map_data$location == "Republic of Korea"] = 'South Korea'

small_map_data$location[small_map_data$location == "United Republic of Tanzania"] ='Tanzania'

small_map_data$location[small_map_data$location == "Bolivia (Plurinational State of)"] ='Bolivia'

small_map_data$location[small_map_data$location == "Venezuela (Bolivarian Republic of)"] ='Venezuela'

small_map_data$location[small_map_data$location == "Czechia"] = 'Czech Republic'

small_map_data$location[small_map_data$location == "Republic of Moldova"] = 'Moldova'

small_map_data$location[small_map_data$location == "Viet Nam"] = 'Vietnam'

small_map_data$location[small_map_data$location == "Lao People's Democratic Republic"] ='Laos'

small_map_data$location[small_map_data$location == "Syrian Arab Republic"] = 'Syria'

small_map_data$location[small_map_data$location == "North Macedonia"] = 'Macedonia'

small_map_data$location[small_map_data$location == "Micronesia (Federated States of)"] ='Micronesia'

small_map_data$location[small_map_data$location == "Macedonia"] = 'North Macedonia'

small_map_data$location[small_map_data$location == "Trinidad and Tobago"] = 'Trinidad'

a <- small_map_data[small_map_data$location == "Trinidad",]

a$location <- 'Tobago'

small_map_data <- rbind(small_map_data,a)

small_map_data$location[small_map_data$location == "Cabo Verde"] = 'Cape Verde'

small_map_data$location[small_map_data$location == "United States Virgin Islands"] ='Virgin Islands'

small_map_data$location[small_map_data$location == "Antigua and Barbuda"] = 'Antigu'

a <- small_map_data[small_map_data$location == "Antigu",]

a$location <- 'Barbuda'

small_map_data <- rbind(small_map_data,a)

small_map_data$location[small_map_data$location == "Saint Kitts and Nevis"] = 'Saint Kitts'

a <- small_map_data[small_map_data$location == "Saint Kitts",]

a$location <- 'Nevis'

small_map_data <- rbind(small_map_data,a)

small_map_data$location[small_map_data$location == "Côte d'Ivoire"] = 'Ivory Coast'

small_map_data$location[small_map_data$location == "Saint Vincent and the Grenadines"] =

'Saint Vincent'

a <- small_map_data[small_map_data$location == "Saint Vincent",]

a$location <- 'Grenadines'

small_map_data <- rbind(small_map_data,a)

small_map_data$location[small_map_data$location == "Eswatini"] = 'Swaziland'

small_map_data$location[small_map_data$location == "Brunei Darussalam"] = 'Brunei'

small_map_data <- full_join(worldData,small_map_data,by = c('region'='location')) %>%

filter(val != "NA")

fig <- ggplot(data=small_map_data)+geom_polygon(aes(x = long, y = lat,group = group,fill=changerate),

colour="black",size=0.5)+theme_bw()+

theme(axis.title.x = element_blank(),

axis.text.x = element_blank(),

axis.ticks.x = element_blank(),axis.text.y = element_blank(),

axis.ticks.y = element_blank(),axis.title.y = element_blank())+

labs(title ="B Change in death cases" )

fxp2<- fig+ labs(x=" ",y="",title="Caribbean and central America")+

coord_cartesian(xlim = c(-92,-60),ylim = c(5,27))+theme(panel.border = element_rect(color='black',fill=NA,size = 0.5))+

theme(legend.position = "none")

fxp3 <- fig+ labs(x=" ",y="",title="Persian Gulf")+

coord_cartesian(xlim = c(45,55),ylim = c(19,31))+theme(panel.border = element_rect(color='black',fill=NA,size = 0.5))+

theme(legend.position = "none")

fxp4 <- fig+ labs(x=" ",y="",title="Balkan Peninsula")+

coord_cartesian(xlim = c(12,32),ylim = c(35,53))+theme(panel.border = element_rect(color='black',fill=NA,size = 0.5))+

theme(legend.position = "none")

fxp5 <- fig+ labs(x=" ",y="",title="Sotheast Asia")+

coord_cartesian(xlim = c(98,123),ylim = c(-10,8))+theme(panel.border = element_rect(color='black',fill=NA,size = 0.5))+

theme(legend.position = "none")

fxp6 <- fig+ labs(x=" ",y="",title="West Africa") +

coord_cartesian(xlim = c(-17,-7),ylim = c(7,20))+theme(panel.border = element_rect(color='black',fill=NA,size = 0.5))+

theme(legend.position = "none")

fxp7 <- fig+ labs(x=" ",y="",title="Eastern \nMediterranean")+

coord_cartesian(xlim = c(32,37),ylim = c(29,35))+theme(panel.border = element_rect(color='black',fill=NA,size = 0.5))+

theme(legend.position = "none")

fxp8 <- fig+ labs(x=" ",y="",title="Northern Europe",size=4) +

coord_cartesian(xlim = c(5,25),ylim = c(48,60))+theme(panel.border = element_rect(color='black',fill=NA,size = 0.5))+

theme(legend.position = "none")

B=(fxp6|fxp7)/fxp8

B

plot<- fig +

(fxp2+fxp3+fxp4+fxp5+B+plot_layout(ncol = 5,widths=c(1.5,1,1.1,1.2,1)))+

plot_layout(ncol = 1,heights = c(9, 3))

plot

ggsave("changenation_death.jpeg",width=16,height=9,dpi=450)

#nation3

small_map_data <- changeold_nem |> filter(measure=="DALYs (Disability-Adjusted Life Years)")

small_map_data$location[small_map_data$location == 'United States of America'] = 'USA'

small_map_data$location[small_map_data$location == 'Russian Federation'] = 'Russia'

small_map_data$location[small_map_data$location == 'United Kingdom'] = 'UK'

small_map_data$location[small_map_data$location == 'Congo'] = 'Republic of Congo'

small_map_data$location[small_map_data$location == "Iran (Islamic Republic of)"] = 'Iran'

small_map_data$location[small_map_data$location == "Democratic People's Republic of Korea"]= 'North Korea'

small_map_data$location[small_map_data$location == "Taiwan (Province of China)"] = 'Taiwan'

small_map_data$location[small_map_data$location == "Republic of Korea"] = 'South Korea'

small_map_data$location[small_map_data$location == "United Republic of Tanzania"] ='Tanzania'

small_map_data$location[small_map_data$location == "Bolivia (Plurinational State of)"] ='Bolivia'

small_map_data$location[small_map_data$location == "Venezuela (Bolivarian Republic of)"] ='Venezuela'

small_map_data$location[small_map_data$location == "Czechia"] = 'Czech Republic'

small_map_data$location[small_map_data$location == "Republic of Moldova"] = 'Moldova'

small_map_data$location[small_map_data$location == "Viet Nam"] = 'Vietnam'

small_map_data$location[small_map_data$location == "Lao People's Democratic Republic"] ='Laos'

small_map_data$location[small_map_data$location == "Syrian Arab Republic"] = 'Syria'

small_map_data$location[small_map_data$location == "North Macedonia"] = 'Macedonia'

small_map_data$location[small_map_data$location == "Micronesia (Federated States of)"] ='Micronesia'

small_map_data$location[small_map_data$location == "Macedonia"] = 'North Macedonia'

small_map_data$location[small_map_data$location == "Trinidad and Tobago"] = 'Trinidad'

a <- small_map_data[small_map_data$location == "Trinidad",]

a$location <- 'Tobago'

small_map_data <- rbind(small_map_data,a)

small_map_data$location[small_map_data$location == "Cabo Verde"] = 'Cape Verde'

small_map_data$location[small_map_data$location == "United States Virgin Islands"] ='Virgin Islands'

small_map_data$location[small_map_data$location == "Antigua and Barbuda"] = 'Antigu'

a <- small_map_data[small_map_data$location == "Antigu",]

a$location <- 'Barbuda'

small_map_data <- rbind(small_map_data,a)

small_map_data$location[small_map_data$location == "Saint Kitts and Nevis"] = 'Saint Kitts'

a <- small_map_data[small_map_data$location == "Saint Kitts",]

a$location <- 'Nevis'

small_map_data <- rbind(small_map_data,a)

small_map_data$location[small_map_data$location == "Côte d'Ivoire"] = 'Ivory Coast'

small_map_data$location[small_map_data$location == "Saint Vincent and the Grenadines"] =

'Saint Vincent'

a <- small_map_data[small_map_data$location == "Saint Vincent",]

a$location <- 'Grenadines'

small_map_data <- rbind(small_map_data,a)

small_map_data$location[small_map_data$location == "Eswatini"] = 'Swaziland'

small_map_data$location[small_map_data$location == "Brunei Darussalam"] = 'Brunei'

small_map_data <- full_join(worldData,small_map_data,by = c('region'='location')) %>%

filter(val != "NA")

fig <- ggplot(data=small_map_data)+geom_polygon(aes(x = long, y = lat,group = group,fill=changerate),

colour="black",size=0.5)+theme_bw()+

theme(axis.title.x = element_blank(),

axis.text.x = element_blank(),

axis.ticks.x = element_blank(),axis.text.y = element_blank(),

axis.ticks.y = element_blank(),axis.title.y = element_blank())+

labs(title ="C Change in DALYs cases" )

fxp2<- fig+ labs(x=" ",y="",title="Caribbean and central America")+

coord_cartesian(xlim = c(-92,-60),ylim = c(5,27))+theme(panel.border = element_rect(color='black',fill=NA,size = 0.5))+

theme(legend.position = "none")

fxp3 <- fig+ labs(x=" ",y="",title="Persian Gulf")+

coord_cartesian(xlim = c(45,55),ylim = c(19,31))+theme(panel.border = element_rect(color='black',fill=NA,size = 0.5))+

theme(legend.position = "none")

fxp4 <- fig+ labs(x=" ",y="",title="Balkan Peninsula")+

coord_cartesian(xlim = c(12,32),ylim = c(35,53))+theme(panel.border = element_rect(color='black',fill=NA,size = 0.5))+

theme(legend.position = "none")

fxp5 <- fig+ labs(x=" ",y="",title="Sotheast Asia")+

coord_cartesian(xlim = c(98,123),ylim = c(-10,8))+theme(panel.border = element_rect(color='black',fill=NA,size = 0.5))+

theme(legend.position = "none")

fxp6 <- fig+ labs(x=" ",y="",title="West Africa") +

coord_cartesian(xlim = c(-17,-7),ylim = c(7,20))+theme(panel.border = element_rect(color='black',fill=NA,size = 0.5))+

theme(legend.position = "none")

fxp7 <- fig+ labs(x=" ",y="",title="Eastern \nMediterranean")+

coord_cartesian(xlim = c(32,37),ylim = c(29,35))+theme(panel.border = element_rect(color='black',fill=NA,size = 0.5))+

theme(legend.position = "none")

fxp8 <- fig+ labs(x=" ",y="",title="Northern Europe",size=4) +

coord_cartesian(xlim = c(5,25),ylim = c(48,60))+theme(panel.border = element_rect(color='black',fill=NA,size = 0.5))+

theme(legend.position = "none")

C=(fxp6|fxp7)/fxp8

C

plot<- fig +

(fxp2+fxp3+fxp4+fxp5+B+plot_layout(ncol = 5,widths=c(1.5,1,1.1,1.2,1)))+

plot_layout(ncol = 1,heights = c(9, 3))

print(plot)

ggsave("DALYs.pdf",plot = plot,width=16,height=9,dpi=450)

###############################################################################################################################################################

##AAPC

df=fread("total.csv",header = T) %>% mutate(measure_name = if_else(measure_name == "DALYs (Disability-Adjusted Life Years)", "DALYs", measure_name)) %>%

filter(year>1989)

seg_apcNew=function(dfx=dfx,labx="xxx",decx=2){

df1=dfx #%>% dplyr::select(year,val)

# Fit the initial linear model

lm_model <- glm((val)~ year, data = df1)

# Fit the segmented model with two breakpoints

fit_segmented_model <- function(model, breakpoints) {

tryCatch({

segmented(model, seg.Z = ~ year, psi = breakpoints)

}, warning = function(w) {

if (grepl("Breakpoint estimate", w$message)) {

cat("Warning: Breakpoint estimate(s) outdistanced to allow finite estimates and st.errs.\n")

NULL

} else {

warning(w)

NULL

}

}, error = function(e) {

cat("Error: Failed to fit the segmented regression model.\n")

NULL

})

}

# Fit the segmented model (you might need to specify the breakpoints if necessary)

# Fit the initial segmented model with breakpoints c(1997, 2005, 2012)

segmented_model <- fit_segmented_model(lm_model, c(1997, 2005, 2012))

# Check if the initial segmented model was successfully fitted

if (!is.null(segmented_model)) {

# Condition 1: If the initial segmented model was successfully fitted

print(1)

} else {

# Condition 2: If the initial segmented model failed, try the alternative breakpoints c(1995, 2000, 2014)

segmented_model <- fit_segmented_model(lm_model, c(1995, 2000, 2014))

if (!is.null(segmented_model)) {

# Condition 3: If the alternative segmented model was successfully fitted

print(2)

} else {

# Condition 4: If both attempts failed, try the second alternative breakpoints c(1990, 2021)

segmented_model <- fit_segmented_model(lm_model, c(1990, 2021))

if (!is.null(segmented_model)) {

# Condition 5: If the second alternative segmented model was successfully fitted

print(3)

} else {

# Condition 6: If all attempts failed, print a message

segmented_model <- fit_segmented_model(lm_model, c( 2021))

if (!is.null(segmented_model)) {

# Condition 5: If the second alternative segmented model was successfully fitted

print(4)

} else {

cat("Failed to fit the segmented regression model.\n")

}

}

}

}

# Check if the segmented model was successfully fitted

# Check if the segmented model was successfully fitted

if (!is.null(segmented_model)) {

# Print the summary of the segmented model

summary(segmented_model)

} else {

cat("Failed to fit the segmented regression model.\n")

}

# Print the estimated breakpoints

plot(segmented_model)

## Calculate the Average Annual Percent Change (AAPC)

aapc_result <- aapc(segmented_model, parm = "year")

print(aapc_result)

df1$fit=as.numeric(segmented_model$fitted.values)

## breakpoints

breakpoints=as.numeric(round(segmented_model$indexU$year))

dft=df1 %>% filter(year %in% breakpoints)

# have to provide estimates for breakpoints.

# after looking a the data,

my.seg <- segmented_model

# display the summary

summary(my.seg)

# get the breakpoints

my.seg$psi

# get the slopes

slope(my.seg)

# get the fitted data

my.fitted <- fitted(my.seg)

my.model <- data.frame(year = df1$year, val = my.fitted)

# plot the fitted model

p1=ggplot() +

geom_line(data=my.model, aes(x = year, y = val),color="red")+

geom_point(data=df1,aes(year,val),color="#7B68EE",size=1)+

geom_vline(xintercept = breakpoints, linetype = "dashed")+

scale_x_continuous(breaks = c(1990,1995,2000,2005,2010,2015,2018,2021),

labels = c(1990,1995,2000,2005,2010,2015,2018,2021))+

# Add breakpoint text annotations

annotate("text", x = breakpoints, y = dft$val, label = as.character(breakpoints),

vjust = -1, hjust = -0.1, color = "black", size = 3.5)+

theme_bw()+

labs(x="Time",y=unique(dfx$measure_name))

# Define the plotting function

plot_segmented_with_points <- function(segmented_model, breakpoints, dfx1) {

# Plot the segmented model

plot(segmented_model, main = "", xlab = "")

# Add vertical dashed lines at the specified years

abline(v = breakpoints, col = "red", lty = 2)

# Adding points from dfx1

points(dfx1$year, dfx1$val, pch = 16, col = "blue", cex = 0.5)

# Update the x-axis and y-axis labels

title(xlab = "x", ylab = "Value")

}

# Call the function to plot

#print(plot_segmented_with_points(segmented_model, breakpoints, dfx1))

# Fit the initial linear model

lm_model <- glm(log(val) ~ year, data = df1)

# Fit the segmented model with two breakpoints

# Initialize a flag to check if segmentation was successful

segmentation_successful <- FALSE

# Try the first segmentation with psi values of 1997, 2005, 2012

try({

my.seg <- segmented(lm_model, seg.Z = ~ year, psi = c(1997, 2005, 2012))

slope_data <- slope(my.seg)$year %>% as.data.frame() %>%

rename("slope" = 1, "low" = 4, "up" = 5)

slope_data$row.names <- rownames(slope_data)

segmentation_successful <- TRUE

}, silent = TRUE)

# If the first segmentation failed, try with psi values of 1990, 2012, 2021

if (!segmentation_successful) {

try({

my.seg <- segmented(lm_model, seg.Z = ~ year, psi = c(1995, 2000, 2014))

slope_data <- slope(my.seg)$year %>% as.data.frame() %>%

rename("slope" = 1, "low" = 4, "up" = 5)

slope_data$row.names <- rownames(slope_data)

segmentation_successful <- TRUE

}, silent = TRUE)

}

# If the second segmentation also failed, try with psi values of 2005, 2012

if (!segmentation_successful) {

my.seg <- segmented(lm_model, seg.Z = ~ year, psi = c(1990, 2021))

slope_data <- slope(my.seg)$year %>% as.data.frame() %>%

rename("slope" = 1, "low" = 4, "up" = 5)

slope_data$row.names <- rownames(slope_data)

}

# Function to calculate APC from slope

# Function to calculate APC from slope

calculate_apc <- function(slope) {

apc <- (exp(slope ) - 1) * 100

return(apc)

}

# Calculate APC for each slope and its confidence intervals

slope_data$APC <- sapply(slope_data$slope, calculate_apc)

slope_data$APC_CI_95_l <- sapply(slope_data$low, calculate_apc)

slope_data$APC_CI_95_u <- sapply(slope_data$up, calculate_apc)

# Function to determine significance

significance_test <- function(lower, upper) {

if (lower > 0 || upper < 0) {

return("*")

} else {

return("")

}

}

# Add significance column

slope_data$p <- mapply(significance_test, slope_data$APC_CI_95_l, slope_data$APC_CI_95_u)

slope_data=slope_data %>%

select(row.names,APC,APC_CI_95_l,APC_CI_95_u,p) %>%

#mutate(APCs=sprintf("%.3f(%.3f,%.3f)",APC, APC_CI_95_l, APC_CI_95_u)) %>%

mutate(APCs= sprintf(paste0("%.", decx, "f(%.", decx, "f,%.", decx, "f)"), APC, APC_CI_95_l, APC_CI_95_u)) %>%

select(row.names,APCs,p)

# Print the results

num=sprintf("AAPC= %.3f(%.3f,%.3f)",aapc_result[1], aapc_result[3], aapc_result[4])

# Print the estimated breakpoints

plot(my.seg)

## Calculate the Average Annual Percent Change (AAPC)

df1$fit=as.numeric(my.seg$fitted.values)

## breakpoints

breakpoints=as.numeric(round(my.seg$indexU$year))

dft=df1 %>% filter(year %in% breakpoints)

# have to provide estimates for breakpoints.

# after looking a the data,

my.seg <- my.seg

# display the summary

# get the breakpoints

my.seg$psi

# get the slopes

slope(my.seg)

# get the fitted data

my.fitted <- fitted(my.seg)

my.model <- data.frame(year = df1$year, val = my.fitted)

generate_time_intervals <- function(start_year, end_year, breakpoints) {

# 将起始年份、拐点和结束年份组合

all_points <- sort(c(start_year, breakpoints, end_year))

# 生成时间区间

time_intervals <- paste0(head(all_points, -1), "-", tail(all_points, -1))

return(time_intervals)

}

# 示例：自动生成时间区间

start_year <- 1990 # 起始年份

end_year <- 2021 # 结束年份

#breakpoints <- c(1995, 2007, 2013) # 动态拐点

# 调用函数生成时间区间

time_intervals <- generate_time_intervals(start_year, end_year, breakpoints)

# 查看结果

print(time_intervals)

slope_data$time <- time_intervals

p2 <- ggplot() +

# 添加拟合线

geom_line(data = my.model, aes(x = year, y = exp(val)), color = "red") +

# 添加原始点

geom_point(data = df1, aes(x = year, y = val), color = "#7B68EE", size = 1) +

# 添加垂直线表示拐点

geom_vline(xintercept = breakpoints, linetype = "dashed") +

# 自定义 X 轴刻度

scale_x_continuous(

breaks = c(1990, 1995, 2000, 2005, 2010, 2015, 2018, 2021),

labels = c(1990, 1995, 2000, 2005, 2010, 2015, 2018, 2021)

) +

# 添加时间区间和 APCs 的注释作为图例

geom_label(

data = slope_data,

aes(

x = c(1993, 2001, 2010, 2017), # 每段时间的中心年份

y = max(exp(my.model$val)), # 注释位置 (顶部偏下)

label = paste0(time, "\nAPC: ", APCs, "", p)

),

fill = "transparent", # 设置背景透明

color = "black",

size = 4.5, # 调整字体大小

fontface = "bold", # 加粗字体

label.size = 0 # 去掉边框

) +

# 添加拐点的注释

annotate(

"text",

x = breakpoints,

y = dft$val,

label = as.character(breakpoints),

vjust = -1,

hjust = -0.1,

color = "blue",

size = 3.5,

fontface = "bold" # 加粗拐点注释字体

) +

# 使用主题

theme_bw() +

# 自定义标签

labs(

x = "Time",

y = unique(dfx$measure_name)

)

print(p2)

# Print the results

return(list(dfx=dfx,aapc=num,p=p2,apcslope=slope_data,plotdata=my.model))

}

for (i in unique(df$location_name)) {print(i)

df1 <- df %>%

filter(location_name==i)

for (m in unique(df1$measure_name)) {print(m)

df2 <- df1 %>%

filter(measure_name == m) %>%

filter(age_id == 197) %>%

filter(metric_name == "Rate") %>%

filter(sex_name == "Female")

seg_apcNew=function(dfx=dfx,labx="xxx",decx=2){

df1=dfx #%>% dplyr::select(year,val)

# Fit the initial linear model

lm_model <- glm((val)~ year, data = df1)

# Fit the segmented model with two breakpoints

fit_segmented_model <- function(model, breakpoints) {

tryCatch({

segmented(model, seg.Z = ~ year, psi = breakpoints)

}, warning = function(w) {

if (grepl("Breakpoint estimate", w$message)) {

cat("Warning: Breakpoint estimate(s) outdistanced to allow finite estimates and st.errs.\n")

NULL

} else {

warning(w)

NULL

}

}, error = function(e) {

cat("Error: Failed to fit the segmented regression model.\n")

NULL

})

}

# Fit the segmented model (you might need to specify the breakpoints if necessary)

# Fit the initial segmented model with breakpoints c(1997, 2005, 2012)

segmented_model <- fit_segmented_model(lm_model, c(1997, 2005, 2012))

# Check if the initial segmented model was successfully fitted

if (!is.null(segmented_model)) {

# Condition 1: If the initial segmented model was successfully fitted

print(1)

} else {

# Condition 2: If the initial segmented model failed, try the alternative breakpoints c(1995, 2000, 2014)

segmented_model <- fit_segmented_model(lm_model, c(1995, 2000, 2014))

if (!is.null(segmented_model)) {

# Condition 3: If the alternative segmented model was successfully fitted

print(2)

} else {

# Condition 4: If both attempts failed, try the second alternative breakpoints c(1990, 2021)

segmented_model <- fit_segmented_model(lm_model, c(1990, 2021))

if (!is.null(segmented_model)) {

# Condition 5: If the second alternative segmented model was successfully fitted

print(3)

} else {

# Condition 6: If all attempts failed, print a message

segmented_model <- fit_segmented_model(lm_model, c( 2021))

if (!is.null(segmented_model)) {

# Condition 5: If the second alternative segmented model was successfully fitted

print(4)

} else {

cat("Failed to fit the segmented regression model.\n")

}

}

}

}

# Check if the segmented model was successfully fitted

# Check if the segmented model was successfully fitted

if (!is.null(segmented_model)) {

# Print the summary of the segmented model

summary(segmented_model)

} else {

cat("Failed to fit the segmented regression model.\n")

}

# Print the estimated breakpoints

plot(segmented_model)

## Calculate the Average Annual Percent Change (AAPC)

aapc_result <- aapc(segmented_model, parm = "year")

print(aapc_result)

df1$fit=as.numeric(segmented_model$fitted.values)

## breakpoints

breakpoints=as.numeric(round(segmented_model$indexU$year))

dft=df1 %>% filter(year %in% breakpoints)

# have to provide estimates for breakpoints.

# after looking a the data,

my.seg <- segmented_model

# display the summary

summary(my.seg)

# get the breakpoints

my.seg$psi

# get the slopes

slope(my.seg)

# get the fitted data

my.fitted <- fitted(my.seg)

my.model <- data.frame(year = df1$year, val = my.fitted)

# plot the fitted model

p1=ggplot() +

geom_line(data=my.model, aes(x = year, y = val),color="red")+

geom_point(data=df1,aes(year,val),color="#7B68EE",size=1)+

geom_vline(xintercept = breakpoints, linetype = "dashed")+

scale_x_continuous(breaks = c(1990,1995,2000,2005,2010,2015,2018,2021),

labels = c(1990,1995,2000,2005,2010,2015,2018,2021))+

# Add breakpoint text annotations

annotate("text", x = breakpoints, y = dft$val, label = as.character(breakpoints),

vjust = -1, hjust = -0.1, color = "black", size = 3.5)+

theme_bw()+

labs(x="Time",y=unique(dfx$measure_name))

# Define the plotting function

plot_segmented_with_points <- function(segmented_model, breakpoints, dfx1) {

# Plot the segmented model

plot(segmented_model, main = "", xlab = "")

# Add vertical dashed lines at the specified years

abline(v = breakpoints, col = "red", lty = 2)

# Adding points from dfx1

points(dfx1$year, dfx1$val, pch = 16, col = "blue", cex = 0.5)

# Update the x-axis and y-axis labels

title(xlab = "x", ylab = "Value")

}

# Call the function to plot

#print(plot_segmented_with_points(segmented_model, breakpoints, dfx1))

# Fit the initial linear model

lm_model <- glm(log(val) ~ year, data = df1)

# Fit the segmented model with two breakpoints

# Initialize a flag to check if segmentation was successful

segmentation_successful <- FALSE

# Try the first segmentation with psi values of 1997, 2005, 2012

try({

my.seg <- segmented(lm_model, seg.Z = ~ year, psi = c(1997, 2005, 2012))

slope_data <- slope(my.seg)$year %>% as.data.frame() %>%

rename("slope" = 1, "low" = 4, "up" = 5)

slope_data$row.names <- rownames(slope_data)

segmentation_successful <- TRUE

}, silent = TRUE)

# If the first segmentation failed, try with psi values of 1990, 2012, 2021

if (!segmentation_successful) {

try({

my.seg <- segmented(lm_model, seg.Z = ~ year, psi = c(1995, 2000, 2014))

slope_data <- slope(my.seg)$year %>% as.data.frame() %>%

rename("slope" = 1, "low" = 4, "up" = 5)

slope_data$row.names <- rownames(slope_data)

segmentation_successful <- TRUE

}, silent = TRUE)

}

# If the second segmentation also failed, try with psi values of 2005, 2012

if (!segmentation_successful) {

my.seg <- segmented(lm_model, seg.Z = ~ year, psi = c(1990, 2021))

slope_data <- slope(my.seg)$year %>% as.data.frame() %>%

rename("slope" = 1, "low" = 4, "up" = 5)

slope_data$row.names <- rownames(slope_data)

}

# Function to calculate APC from slope

# Function to calculate APC from slope

calculate_apc <- function(slope) {

apc <- (exp(slope ) - 1) * 100

return(apc)

}

# Calculate APC for each slope and its confidence intervals

slope_data$APC <- sapply(slope_data$slope, calculate_apc)

slope_data$APC_CI_95_l <- sapply(slope_data$low, calculate_apc)

slope_data$APC_CI_95_u <- sapply(slope_data$up, calculate_apc)

# Function to determine significance

significance_test <- function(lower, upper) {

if (lower > 0 || upper < 0) {

return("*")

} else {

return("")

}

}

# Add significance column

slope_data$p <- mapply(significance_test, slope_data$APC_CI_95_l, slope_data$APC_CI_95_u)

slope_data=slope_data %>%

select(row.names,APC,APC_CI_95_l,APC_CI_95_u,p) %>%

#mutate(APCs=sprintf("%.3f(%.3f,%.3f)",APC, APC_CI_95_l, APC_CI_95_u)) %>%

mutate(APCs= sprintf(paste0("%.", decx, "f(%.", decx, "f,%.", decx, "f)"), APC, APC_CI_95_l, APC_CI_95_u)) %>%

select(row.names,APCs,p)

# Print the results

num=sprintf("AAPC= %.3f(%.3f,%.3f)",aapc_result[1], aapc_result[3], aapc_result[4])

# Print the estimated breakpoints

plot(my.seg)

## Calculate the Average Annual Percent Change (AAPC)

df1$fit=as.numeric(my.seg$fitted.values)

## breakpoints

breakpoints=as.numeric(round(my.seg$indexU$year))

dft=df1 %>% filter(year %in% breakpoints)

# have to provide estimates for breakpoints.

# after looking a the data,

my.seg <- my.seg

# display the summary

# get the breakpoints

my.seg$psi

# get the slopes

slope(my.seg)

# get the fitted data

my.fitted <- fitted(my.seg)

my.model <- data.frame(year = df1$year, val = my.fitted)

generate_time_intervals <- function(start_year, end_year, breakpoints) {

# 将起始年份、拐点和结束年份组合

all_points <- sort(c(start_year, breakpoints, end_year))

# 生成时间区间

time_intervals <- paste0(head(all_points, -1), "-", tail(all_points, -1))

return(time_intervals)

}

# 示例：自动生成时间区间

start_year <- 1990 # 起始年份

end_year <- 2021 # 结束年份

#breakpoints <- c(1995, 2007, 2013) # 动态拐点

# 调用函数生成时间区间

time_intervals <- generate_time_intervals(start_year, end_year, breakpoints)

# 查看结果

print(time_intervals)

slope_data$time <- time_intervals

p2 <- ggplot() +

# 添加拟合线

geom_line(data = my.model, aes(x = year, y = exp(val)), color = "red") +

# 添加原始点

geom_point(data = df1, aes(x = year, y = val), color = "#7B68EE", size = 1) +

# 添加垂直线表示拐点

geom_vline(xintercept = breakpoints, linetype = "dashed") +

# 自定义 X 轴刻度

scale_x_continuous(

breaks = c(1990, 1995, 2000, 2005, 2010, 2015, 2018, 2021),

labels = c(1990, 1995, 2000, 2005, 2010, 2015, 2018, 2021)

) +

# 添加时间区间和 APCs 的注释作为图例

geom_label(

data = slope_data,

aes(

x = c(1993, 2001, 2010, 2017), # 每段时间的中心年份

y = max(exp(my.model$val)), # 注释位置 (顶部偏下)

label = paste0(time, "\nAPC: ", APCs, "", p)

),

fill = "transparent", # 设置背景透明

color = "black",

size = 4.5, # 调整字体大小

fontface = "bold", # 加粗字体

label.size = 0 # 去掉边框

) +

# 添加拐点的注释

annotate(

"text",

x = breakpoints,

y = dft$val,

label = as.character(breakpoints),

vjust = -1,

hjust = -0.1,

color = "blue",

size = 3.5,

fontface = "bold" # 加粗拐点注释字体

) +

# 使用主题

theme_bw() +

# 自定义标签

labs(

x = "Time",

y = unique(dfx$measure_name)

)

print(p2)

# Print the results

return(list(dfx=dfx,aapc=num,p=p2,apcslope=slope_data,plotdata=my.model))

}

xa <- seg_apcNew(dfx=df2,labx="x",decx=2)

ggsave(paste0("Fig-AAPC-",i,"-",m,".pdf"), plot = xa$p, width = 12, height = 8)

write.csv(xa$apcslope, paste0("Fig-AAPC-",i,"-",m,".csv"), row.names = FALSE)

write.csv(df2, paste0("Fig-AAPC-",i,"-",m,"-data.csv"), row.names = FALSE)

}}

###############################################################################################################################################################

##APC web tools

df <- read.csv("total.csv") %>%

filter(year>1989)

for (i in unique(df$measure_name)) {print(i)

dfx1 <- df %>%

filter(measure_name == i) %>%

filter(location_name=="region") %>%

filter(sex_name=="Female") %>%

filter(metric_name =="Rate") %>%

filter(age_id %in% c(1, 5:20, 30, 31, 32, 235))

dfx2 <- df %>%

filter(measure_name == i) %>%

filter(location_name=="region") %>%

filter(sex_name=="Female") %>%

filter(metric_name =="Number") %>%

filter(age_id %in% c(1, 5:20, 30, 31, 32, 235))

dfxrate=dfx1

dfxnumber=dfx2

CI=T

#source("apc.R")

######## ######## ######## ######## ########

## Table1

df1=dfxrate

#print(dim(df1))

# Create the xage dataframe

xage <- data.frame(

age_id = c(1, 6, 7, 8, 9, 10, 11, 12, 13, 14, 15, 16, 17, 18, 19, 20, 30, 31, 32, 235),

age_name = c("<5 years", "5-9 years", "10-14 years", "15-19 years", "20-24 years",

"25-29 years", "30-34 years", "35-39 years", "40-44 years",

"45-49 years", "50-54 years", "55-59 years", "60-64 years",

"65-69 years", "70-74 years", "75-79 years", "80-84 years",

"85-89 years", "90-94 years", "95+ years"),

id = c(12, 13, 16, 20, 24, 27, 30, 31, 32, 33, 34, 37, 39, 40, 43, 45, 48, 50, 52, 53),

age = c(0, 5, 10, 15, 20, 25, 30, 35, 40, 45, 50, 55, 60, 65, 70, 75, 80, 85, 90, 95),

mid = c(2, 7, 12, 17, 22, 27, 32, 37, 42, 47, 52, 57, 62, 67, 72, 77, 82, 87, 92, 97)

)

mid=df1 %>% dplyr::select(age_name) %>% unique() %>%

left_join(.,xage) %>% arrange(id)

mid

dfp1=df1 %>% dplyr::select(age_id,age_name,year,val) %>%

mutate(age_name=factor(age_name,level=xage$age_name)) %>%

arrange(age_id,year) %>%

mutate(age=rep(mid$mid,each=32),coh=year-age)

#x=dfp1 %>% filter(age_name=="80-84 years")

p1 = ggplot(dfp1) +

geom_line(aes(x = coh, y = val, group = age_name, color = age_name),linewidth = 1) +

labs(

title = paste0("Cohort-specific rates by age group "),

color = "",

x = "Birth Cohort",

y = paste0("Rate of ", i, " (per 100 000)")

) +

guides(

color = guide_legend(ncol = 1, byrow = TRUE)

) +

theme_classic() +

scale_x_continuous(

breaks = seq(1890, max(dfp1$coh, na.rm = TRUE), by = 10), # x轴刻度从1890开始，每隔10显示一个刻度

limits = c(1890, max(dfp1$coh, na.rm = TRUE)) # 限制x轴范围从1890到coh的最大值

) +

theme(

plot.title = element_text(size = 18, face = "bold", hjust = 0.5), # 标题字体放大加粗

legend.position = "right",

legend.title = element_text(size = 14, face = "bold"), # 图例标题字体放大加粗

legend.text = element_text(size = 12, face = "bold"), # 图例内容字体放大加粗

axis.title.x = element_text(size = 14, face = "bold"), # x轴标题字体放大加粗

axis.title.y = element_text(size = 14, face = "bold"), # y轴标题字体放大加粗

axis.text.x = element_text(size = 12, face = "bold",angle = 45,hjust = 1), # x轴刻度字体放大加粗

axis.text.y = element_text(size = 12, face = "bold") # y轴刻度字体放大加粗

)

print(p1)

######## ######## ######## ######## ########

## Table2

df_rate = df1 %>%

dplyr::select(location_name,age_name,year,val) %>%

filter(year %in% seq(1994,2021,5)) %>%

arrange(year) %>%

spread(year,val)

b=df_rate %>% left_join(.,xage) %>%

arrange(id) %>%

dplyr::select( age_name,`1994`,`1999`, `2004`, `2009`,`2014`,`2019`)

dfp2=b %>% purrr::set_names("age_name","1992-1996","1997-2001",

"2002-2006","2007-2011","2012-2016",

"2017-2021") %>%

mutate(age=rep(mid$mid+0.5,1)) %>%

gather("Period","rate",-age_name,-age)

#plot

p2 = ggplot(dfp2) +

geom_line(aes(x = age, y = rate, group = Period, color = Period),linewidth = 1) +

labs(

title = paste0("Age-specific rates by period "),

color = "",

x = "Age group",

y = paste0("Rate of ", i, " (per 100 000)")

) +

theme_classic() +

theme(

plot.title = element_text(size = 18, face = "bold", hjust = 0.5), # 标题字体放大加粗

legend.title = element_text(size = 14, face = "bold"), # 图例标题字体放大加粗

legend.text = element_text(size = 12, face = "bold"), # 图例内容字体放大加粗

axis.title.x = element_text(size = 14, face = "bold"), # x轴标题字体放大加粗

axis.title.y = element_text(size = 14, face = "bold"), # y轴标题字体放大加粗

axis.text.x = element_text(size = 12, face = "bold"), # x轴刻度字体放大加粗

axis.text.y = element_text(size = 12, face = "bold") # y轴刻度字体放大加粗

)

print(p2)

df1=dfxnumber

df_rate = df1 %>%

dplyr::select(location_name,age_name,year,val) %>%

filter(year %in% seq(1994,2021,5)) %>%

arrange(year) %>%

spread(year,val)

b=df_rate %>% left_join(.,xage) %>% arrange(id) %>%

dplyr::select( age_name,`1994`,`1999`, `2004`, `2009`,`2014`,`2019`)

midage=b %>% dplyr::select(age_name) %>% unique() %>%

left_join(.,mid)

dfpop=population %>% dplyr::filter(location_id %in% unique(df1$location_id) ) %>%

#filter(cause_name%in% input$tablecause_name10) %>%

filter(sex_name %in% unique(df1$sex_name)) %>%

filter(metric_name %in% "Number") %>%

filter( age_id %in% midage$age_id) %>%

filter(year %in% seq(1994,2021,5)) %>%

dplyr::select(age_id,age_name,year,val) %>%

spread(year,val) %>%

separate(age_name,sep=' ', c("age_name")) %>% as.data.frame()

dfpop1=dfpop %>% dplyr::select(-1,-2)

##### binds five country

apc_list=c();

df_CR=c()

df_PR=c()

df_LongAge=c()

df_local=c()

df_net=c()

df_rate = b

#df_rate=df_rate[3:15,]

#print(df_rate)

#print( dim(df_rate))

a=as.matrix(df_rate[,-1]);colnames(a) <- NULL

popx=as.matrix(dfpop1);

#print(dim(popx))

x=dim(a)[1];y=dim(a)[2]

a=matrix(a,nc=y,nr=x)

# apc data form

test=list(

events=a,

offset=popx,

offset_tick=1,

ages=c(mid$age, max(mid$age)+5),

periods=seq(1992,2022,5)

)

# APC model

df_apc=apc2(test)

# APC model

# CR

cr=df_apc$CohortRR %>% as.data.frame() %>%

purrr::set_names("age","val","low","up") %>%

mutate(type="Cohort RR")

# PR

pr=df_apc$PeriodRR %>% as.data.frame() %>%

purrr::set_names("age","val","low","up") %>%

mutate(type="Period RR")

# longitudinal age curves

la=df_apc$LongAge %>% as.data.frame() %>%

purrr::set_names("age","val","low","up") %>%

mutate(type="Age effect")

# local drift

ld=df_apc$LocalDrifts %>% as.data.frame() %>%

purrr::set_names("age","val","low","up") %>%

mutate(type="Local Drifts")

# net drift

nd=df_apc$NetDrift %>% as.data.frame() %>%

purrr::set_names("val","low","up") %>%

mutate(age=1,.before=1) %>%

mutate(type="Net drift")

# bind

dfxall=rbind(la,cr,pr,ld,nd)

dfxall$sex_name= unique(df1$sex_name)

dfxall$location_name= unique(df1$location_name)

dfxall$location_id= unique(df1$location_id)

##############

# local drift

p3 = ggplot(ld, aes(x = age, y = val)) +

geom_line(aes(x = age, y = val),linewidth = 1) +

geom_point(aes(x = age, y = val)) +

geom_hline(data = nd, aes(yintercept = val, linetype = 'dotted'),

linetype = "dashed", color = "pink") +

# geom_ribbon(aes(ymin = low, ymax = up), fill = "#FF8C00", alpha = 0.3) +

scale_x_continuous(name = "Age", breaks = breaks_width(5)) +

labs(

title = paste0("Net drifts & Local drifts"),

color = "",

y = "Percent per year",

x = "Age"

) +

theme_classic() +

theme(

plot.title = element_text(size = 18, face = "bold", hjust = 0.5), # 标题字体放大加粗

legend.title = element_text(size = 14, face = "bold"), # 图例标题字体放大加粗

legend.text = element_text(size = 12, face = "bold"), # 图例内容字体放大加粗

axis.title.x = element_text(size = 14, face = "bold"), # x轴标题字体放大加粗

axis.title.y = element_text(size = 14, face = "bold"), # y轴标题字体放大加粗

axis.text.x = element_text(size = 12, face = "bold"), # x轴刻度字体放大加粗

axis.text.y = element_text(size = 12, face = "bold") # y轴刻度字体放大加粗

)

print(p3)

if (CI) {

p3= p3+ geom_ribbon(aes(ymin = low, ymax = up), fill = "#FFC0CB", alpha = 0.3)

} else{

p3

}

##############

# longitudinal age curves

p4 = ggplot(la, aes(x = age, y = val)) +

geom_line(aes(x = age, y = val),linewidth = 1) +

geom_point(aes(x = age, y = val)) +

scale_x_continuous(name = "Age", breaks = breaks_width(5)) +

labs(

title = paste("Age effects"),

color = "",

y = paste0("Rate of ", i, " (per 100 000 person-years)"),

x = "Age"

) +

theme_classic() +

theme(

plot.title = element_text(size = 18, face = "bold", hjust = 0.5), # 标题字体放大加粗

legend.title = element_text(size = 14, face = "bold"), # 图例标题字体放大加粗

legend.text = element_text(size = 12, face = "bold"), # 图例内容字体放大加粗

axis.title.x = element_text(size = 14, face = "bold"), # x轴标题字体放大加粗

axis.title.y = element_text(size = 14, face = "bold"), # y轴标题字体放大加粗

axis.text.x = element_text(size = 12, face = "bold"), # x轴刻度字体放大加粗

axis.text.y = element_text(size = 12, face = "bold") # y轴刻度字体放大加粗

)

print(p4)

if (CI) {

p4= p4+ geom_ribbon(aes(ymin = low, ymax = up), fill = "#FF8C00", alpha = 0.3)

} else{

p4

}

##############

# period effects

p5 = ggplot(pr, aes(x = age, y = val)) +

geom_line(aes(x = age, y = val),linewidth = 1) +

geom_point(aes(x = age, y = val)) +

geom_hline(data = df_net, aes(yintercept = 1), linetype = "dashed") +

# geom_ribbon(aes(ymin = low, ymax = up), fill = "#20B2AA", alpha = 0.3) +

labs(

title = paste("Period effects"),

color = "",

y = "Rate Ratio",

x = "Period"

) +

theme_classic() +

theme(

plot.title = element_text(size = 18, face = "bold", hjust = 0.5), # 标题字体放大加粗

legend.title = element_text(size = 14, face = "bold"), # 图例标题字体放大加粗

legend.text = element_text(size = 12, face = "bold"), # 图例内容字体放大加粗

axis.title.x = element_text(size = 14, face = "bold"), # x轴标题字体放大加粗

axis.title.y = element_text(size = 14, face = "bold"), # y轴标题字体放大加粗

axis.text.x = element_text(size = 12, face = "bold"), # x轴刻度字体放大加粗

axis.text.y = element_text(size = 12, face = "bold") # y轴刻度字体放大加粗

)

print(p5)

if (CI) {

p5= p5+ geom_ribbon(aes(ymin = low, ymax = up), fill = "#20B2AA", alpha = 0.3)

} else{

p5

}

##############

# cohort effects

p6 = ggplot(cr, aes(x = age, y = val)) +

geom_line(aes(x = age, y = val),linewidth = 1) +

geom_point(aes(x = age, y = val)) +

geom_hline(data = df_net, aes(yintercept = 1), linetype = "dashed") +

# geom_ribbon(aes(ymin = low, ymax = up), fill = "#7B68EE", alpha = 0.3) +

scale_x_continuous(name = "Cohort", breaks = breaks_width(10)) +

labs(

title = paste("Cohort effects"),

color = "",

y = "Rate Ratio",

x = "Cohort"

) +

theme_classic() +

theme(

plot.title = element_text(size = 18, face = "bold", hjust = 0.5), # 标题字体放大加粗

legend.title = element_text(size = 14, face = "bold"), # 图例标题字体放大加粗

legend.text = element_text(size = 12, face = "bold"), # 图例内容字体放大加粗

axis.title.x = element_text(size = 14, face = "bold"), # x轴标题字体放大加粗

axis.title.y = element_text(size = 14, face = "bold"), # y轴标题字体放大加粗

axis.text.x = element_text(size = 12, face = "bold"), # x轴刻度字体放大加粗

axis.text.y = element_text(size = 12, face = "bold") # y轴刻度字体放大加粗

)

print(p6)

if (CI) {

p6= p6+ geom_ribbon(aes(ymin = low, ymax = up), fill = "#7B68EE", alpha = 0.3)

} else{

p6

}

library(ggpubr)

p=ggarrange(p1,p2,p3,p4,p5,p6,nrow=2,ncol=3,labels="AUTO")

pp1=ggarrange(p2,p1,nrow=1,ncol=2,labels="AUTO")

pp2=ggarrange(p3,p4,p5,p6,nrow=2,ncol=2,labels="AUTO")

write.csv(dfp1,paste0("region",i,"-A.csv"))

write.csv(dfp2,paste0("region",i,"-B.csv"))

write.csv(ld,paste0("region",i,"-C.csv"))

write.csv(la,paste0("region",i,"-D.csv"))

write.csv(pr,paste0("region",i,"-E.csv"))

write.csv(cr,paste0("region",i,"-F.csv"))

ggsave(paste0("region",i,".pdf"),plot = p,width = 20,height = 16)

}

###############################################################################################################################################################

##BAPC

library(BAPC)

library(openxlsx)

library(INLA)

library(nordpred)

library(reshape)

library(data.table)

library(tidyr)

library(tidyverse)

library(epitools)

library(ggplot2)

#inla.upgrade() # for the stable version

# 1990-2019年人口学数据

# 发病数据需要的年龄分层

age1 <- c("<5 years","5-9 years","10-14 years","15-19 years","20-24 years",

"25-29 years","30-34 years","35-39 years","40-44 years","45-49 years",

"50-54 years","55-59 years","60-64 years","65-69 years","70-74 years",

"75-79 years","80-84","85-89","90-94","95+ years") ###20个年龄组

#### 调取标准人口百分比用

ages_2 <- c("<1 year","1 to 4", "5 to 9","10 to 14", "15 to 19","20 to 24", "25 to 29",

"30 to 34", "35 to 39", "40 to 44", "45 to 49", "50 to 54", "55 to 59",

"60 to 64", "65 to 69", "70 to 74", "75 to 79", "80 to 84", "85 to 89",

"90 to 94", "95 plus")

#### 预测的年龄结构

ages_3 <- c("0 to 4", "5 to 9","10 to 14", "15 to 19","20 to 24", "25 to 29",

"30 to 34", "35 to 39", "40 to 44", "45 to 49", "50 to 54", "55 to 59",

"60 to 64", "65 to 69", "70 to 74", "75 to 79", "80 to 84", "85 to 89",

"90 to 94", "95 plus")

# 标准年龄结构数据age_stand

age_stand <- read.csv("age_stand.csv")

#标准构成比

wstand <- c(age_stand$std_population[1:2] %>% as.numeric() %>% sum(),

age_stand$std_population[3:21] %>% as.numeric())/sum(age_stand$std_population[1:21])

wstand

sum(wstand)

IBD_china <- read.csv('data.csv')

########Both#######

####Incidence####

#####先处理发生人数数据#####

IBD_in_both<- subset(IBD_china,

(IBD_china$age_name %in% age1 ) &

IBD_china$sex_name=="Both"&

IBD_china$location_name=='Global'&

IBD_china$metric_name== 'Number' &

IBD_china$measure_name=='Incidence') #这些指标可以改

unique(IBD_in_both$age_name)

IBD_in_both$age_name<-gsub(" years","",IBD_in_both$age_name)

IBD_in_both$age_name <- factor(IBD_in_both$age_name, levels = c("<5", "5-9", "10-14", "15-19",

"20-24", "25-29", "30-34", "35-39", "40-44", "45-49", "50-54",

"55-59", "60-64", "65-69", "70-74", "75-79", "80-84", "85-89",

"90-94", "95+"))

# 提取数据Measure_name,age_name,year,val

IBD_in_both <- IBD_in_both[,c("measure_name", "age_name","year","val")]

#长转宽

IBD_in_both_n <- reshape2::dcast(data=IBD_in_both, year ~ age_name, value.var="val")

rownames(IBD_in_both_n) <- IBD_in_both_n$year

IBD_in_both_n <- IBD_in_both_n[,-1]

#IBD_in_both_n <- apply(IBD_in_both_n,c(1,2),as.integer) %>% as.data.frame

####含义####

IBD_in_both_n <- apply(IBD_in_both_n,c(1,2),round) %>% as.data.frame

####人口数据#####

###人口数据

age2 <- c("<5 years","5-9 years","10-14 years","15-19 years","20-24 years",

"25-29 years","30-34 years","35-39 years","40-44 years","45-49 years",

"50-54 years","55-59 years","60-64 years","65-69 years","70-74 years",

"75-79 years","80-84 years","85-89 years","90-94 years","95+ years") ###20个年龄

var_name <- c("location_name", "sex_name", "year", "age_name", "val")

GBD_population <- data.frame()

#names(GBD_population) = var_name

GBD_population <- data.frame()

#population<-data.frame()

for(k in 1:length(fileName)){

data = read.csv(file = paste(path,fileName[k],sep = "\\"),

header = T,stringsAsFactors = F)

GBD_population=rbind(GBD_population,data)

}

GBD_population<-GBD_population%>% dplyr::select(var_name) %>%

filter(location_name %in% 'China' & age_name %in% age2 )

# write.csv(GBD_population,file = "GBD_population.csv")

# GBD_population<-read.csv("GBD_population.csv")

GBD_population$age_name<-gsub(" years","",GBD_population$age_name)

#GBD_population <- GBD_population[!duplicated(GBD_population),]

#GBD_Both_population<- subset(GBD_population,GBD_population$sex_name =="Both")

#GBD_Female_population<- subset(GBD_population,GBD_population$sex_name =="Female")

GBD_Male_population<- subset(GBD_population,GBD_population$sex_name =="Male")

###### 2020-2030年人口学数据#####

prediction_var_name <- c("location_name", "sex", "year_id", "age_group_name", "val")

GBD_population_prediction <- fread("IHME_POP_2017_2100_POP_REFERENCE_Y2020M05D01.csv") %>%

dplyr::select(prediction_var_name) %>%

dplyr::filter(location_name %in% 'China' & year_id %in% 2022:2035

& sex %in% "Male")

unique(GBD_population_prediction$age_group_name)

## 由于预测人口数据没有<1的年龄数据，因此我们需要进行转换，将Early Neonatal,Late Neonatal, Post Neonatal,合并成<1的年龄数据

## 筛选`Early Neonatal`,`Late Neonatal`, `Post Neonatal`数据并根据location_name,sex,year_id 进行分组后对3个年龄的val值相加得到<1 year的人口学数据

## 同时加上1-4岁的数据，组成<5岁的人口数据

GBD_5year <- GBD_population_prediction %>%

filter(age_group_name %in% c("Early Neonatal","Late Neonatal", "Post Neonatal","1 to 4")) %>%

group_by(location_name,sex,year_id) %>%

summarise(val=sum(val)) %>%

mutate(age_group_name="<5")

## 再将 "Early Neonatal","Late Neonatal", "Post Neonatal","1 to 4"数据去除,加上<5 year的人口学数据

GBD_population_prediction <- GBD_population_prediction %>% filter(!(age_group_name %in% c("Early Neonatal","Late Neonatal", "Post Neonatal","All Ages","1 to 4"))) %>%

rbind(GBD_5year)

names(GBD_population_prediction)[names(GBD_population_prediction) == 'age_group_name'] <- 'age_name'

GBD_population_prediction$age_name<-gsub(" to ","-",GBD_population_prediction$age_name)

GBD_population_prediction$age_name<-gsub(" plus","+",GBD_population_prediction$age_name)

unique(GBD_population_prediction$age_name)

colnames(GBD_population_prediction)<-var_name

#####合并人口学数据1990-2036#####

GBD <- rbind(GBD_Male_population, GBD_population_prediction)

GBD$age_name<-factor(GBD$age_name, levels = c("<5", "5-9", "10-14", "15-19",

"20-24", "25-29", "30-34", "35-39", "40-44", "45-49", "50-54",

"55-59", "60-64", "65-69", "70-74", "75-79", "80-84", "85-89",

"90-94", "95+"))

unique(GBD$age_name)

# 整理人口学数据变成BAPC能够识别的数据形式

GBD_China_Male <- subset(GBD,location_name=="Global" & sex_name=="Both")

GBD_China_Male$age_name<-factor(GBD_China_Male$age_name, levels = c("<5", "5-9", "10-14", "15-19",

"20-24", "25-29", "30-34", "35-39", "40-44", "45-49", "50-54",

"55-59", "60-64", "65-69", "70-74", "75-79", "80-84", "85-89",

"90-94", "95+"))

GBD_China_Male_n <- dcast(data=GBD_China_Male, year~age_name, value.var=c("val")) %>% as.data.frame()

#改行名

rownames(GBD_China_Male_n) <- GBD_China_Male_n$year

GBD_China_Male_n <- GBD_China_Male_n[,-1]

GBD_China_Male_n <- apply(GBD_China_Male_n, c(1,2), as.numeric) %>% as.data.frame()

GBD_China_Male_n <- apply(GBD_China_Male_n, c(1,2), round) %>% as.data.frame()

# 补充没有发病人数数据的年份

IBD_pro <- matrix(data=NA, nrow=2035-2021, ncol=ncol(GBD_China_Male_n)) %>% as.data.frame()

rownames(IBD_pro) <- seq(2022, 2035, 1)

colnames(IBD_pro) <- names(IBD_in_both_n)

IBD_pro_n <- rbind(IBD_in_both_n, IBD_pro)

IBD_pro_n <- apply(IBD_pro_n, c(1,2), as.numeric) %>% as.data.frame()

IBD_pro_n <- apply(IBD_pro_n, c(1,2), round) %>% as.data.frame()

require(INLA)

#sum(wstand)

# 模型预测

diabetes3_input <- APCList(IBD_pro_n, GBD_China_Male_n, gf=5)##gf为年份间隔

diabetes3_bapc_result <- BAPC(diabetes3_input, predict=list(npredict=15, retro=T), secondDiff=FALSE, stdweight=wstand, verbose=F)

p1<-plotBAPC(diabetes3_bapc_result, scale=10^5, type = 'ageStdRate', showdata = TRUE)

#提取数据

Both_de =data.frame(diabetes3_bapc_result@agestd.rate)

###############################################################################################################################################################

####分解解析

## Decomposition

library(dplyr)

library(data.table)

library(purrr)

library(tidyr)

library(ggplot2)

library(ggsci)

## 读取疾病数据

IBD_Global <- read.csv('data.csv')

unique(IBD_Global$age_name)

#age1对应IBD——Global数据和age2对应总人口数据

age1 <- c("<5 years","5-9 years","10-14 years","15-19 years","20-24 years",

"25-29 years","30-34 years","35-39 years","40-44 years","45-49 years",

"50-54 years","55-59 years","60-64 years","65-69 years","70-74 years",

"75-79 years","80-84","85-89","90-94","95+ years") ###20个年龄组

age2 <- c("<5 years","5-9 years","10-14 years","15-19 years","20-24 years",

"25-29 years","30-34 years","35-39 years","40-44 years","45-49 years",

"50-54 years","55-59 years","60-64 years","65-69 years","70-74 years",

"75-79 years","80-84 years","85-89 years","90-94 years","95+ years") ###20个年龄

var_name <- c("location_name","sex_name","year","age_name","val")

## sub函数：将年龄的label,比如"15-19 years"转换成“15 to 19”,以匹配人口学数据和疾病数据

IBD_in_both<- subset(IBD_Global,

(IBD_Global$age_name %in% age1 ) &

IBD_Global$location_name=='Global')

IBD_in_both$age_name<-gsub(" years","",IBD_in_both$age_name)

IBD_in_both$age_name <- factor(IBD_in_both$age_name, levels = c("<5", "5-9", "10-14", "15-19",

"20-24", "25-29", "30-34", "35-39", "40-44", "45-49", "50-54",

"55-59", "60-64", "65-69", "70-74", "75-79", "80-84", "85-89",

"90-94", "95+"))

var_name <- c("location_name","sex_name","year","age_id","age_name","val")

population <- data.frame()

#population<-data.frame()

for(k in 1:length(fileName)){

data = read.csv(file = paste(path,fileName[k],sep = "\\"),

header = T,stringsAsFactors = F)

population=rbind(population,data)

}

population<-population%>% dplyr::select("location_name","sex_name","year","age_name","val") %>%

filter(location_name %in% 'Global' & age_name %in% age2)

###

population$age_name<-gsub(" years","",population$age_name)

population$age_name <- factor(population$age_name, levels = c("<5", "5-9", "10-14", "15-19",

"20-24", "25-29", "30-34", "35-39", "40-44", "45-49", "50-54",

"55-59", "60-64", "65-69", "70-74", "75-79", "80-84", "85-89",

"90-94", "95+"))

population<-subset(population,population$year==1990|population$year==2021)

#str(population)

head(population)

#计算1990年全人群的年龄构成

pop1<-subset(population,population$year==1990 & population$sex_name == "Both")

tot1990<-sum(pop1$val)

pop1$percent <- pop1$val/tot1990

#计算2021年全人群的年龄构成

pop2<-subset(population,population$year==2021 & population$sex_name == "Both")

tot2021<-sum(pop2$val)

pop2$percent <- pop2$val/tot2021

#提取分析所用数据

### a代表年龄占比，p代表总人群数，将数据提取出来

a_1990 <- pop1$percent

a_2021 <- pop2$percent

p_1990 <- tot1990

p_2021 <- tot2021

## 获取率的数据

case_1990 <- IBD_in_both %>% filter(year == 1990 &

sex_name == "Both" &

location_name == "Global" &

metric_name == 'Rate' &

measure_name == 'Incidence')

case_2021 <- IBD_in_both %>% filter(year == 2021 &

location_name == 'Global' &

sex_name == "Both" &

metric_name == 'Rate' &

measure_name == 'Incidence')

r_1990 <- as.numeric(case_1990$val)/10^5 ## 单位转换

r_2021 <- as.numeric(case_2021$val)/10^5 ## 单位转换

##### 根据公式计算

a_effect <- round((sum(a_2021*p_1990*r_1990) + sum(a_2021*p_2021*r_2021))/3 +

(sum(a_2021*p_1990*r_2021) + sum(a_2021*p_2021*r_1990))/6 -

(sum(a_1990*p_1990*r_1990) + sum(a_1990*p_2021*r_2021))/3 -

(sum(a_1990*p_1990*r_2021) + sum(a_1990*p_2021*r_1990))/6,3)

p_effect <- round((sum(a_1990*p_2021*r_1990) + sum(a_2021*p_2021*r_2021))/3 +

(sum(a_1990*p_2021*r_2021) + sum(a_2021*p_2021*r_1990))/6 -

(sum(a_1990*p_1990*r_1990) + sum(a_2021*p_1990*r_2021))/3 -

(sum(a_1990*p_1990*r_2021) + sum(a_2021*p_1990*r_1990))/6,3)

r_effect <- round((sum(a_1990*p_1990*r_2021) + sum(a_2021*p_2021*r_2021))/3 +

(sum(a_1990*p_2021*r_2021) + sum(a_2021*p_1990*r_2021))/6 -

(sum(a_1990*p_1990*r_1990) + sum(a_2021*p_2021*r_1990))/3 -

(sum(a_1990*p_2021*r_1990) + sum(a_2021*p_1990*r_1990))/6,3)

overll_differ <- round(a_effect + p_effect + r_effect,2)

a_percent <- round(a_effect/overll_differ*100,2)

p_percent <- round(p_effect/overll_differ*100,2)

r_percent <- round(r_effect/overll_differ*100,2)

#数据合并，形成一个数据集

temp <- c("Both",overll_differ,a_effect,p_effect,r_effect,a_percent,

p_percent,r_percent) %>% t() %>% as.data.frame()

#生成表头

decomposition_name<-c('sex_name','overll_difference','a_effect','p_effect','r_effect','a_percent',

'p_percent','r_percent')

names(temp) <- decomposition_name

#计算1990年全人群的年龄构成

pop1<-subset(population,population$year==1990 & population$sex_name == "Both")

tot1990<-sum(pop1$val)

pop1$percent <- pop1$val/tot1990

#计算2021年全人群的年龄构成

pop2<-subset(population,population$year==2021 & population$sex_name == "Both")

tot2021<-sum(pop2$val)

pop2$percent <- pop2$val/tot2021

#提取分析所用数据

### a代表年龄占比，p代表总人群数，将数据提取出来

a_1990 <- pop1$percent

a_2021 <- pop2$percent

p_1990 <- tot1990

p_2021 <- tot2021

## 获取率的数据

case_1990 <- IBD_in_both %>% filter(year == 1990 &

sex_name == "Both" &

location_name == "Global" &

metric_name == 'Rate' &

measure_name == 'Incidence')

case_2021 <- IBD_in_both %>% filter(year == 2021 &

location_name == 'Global' &

sex_name == "Both" &

metric_name == 'Rate' &

measure_name == 'Incidence')

r_1990 <- as.numeric(case_1990$val)/10^5 ## 单位转换

r_2021 <- as.numeric(case_2021$val)/10^5 ## 单位转换

##### 根据公式计算

a_effect <- round((sum(a_2021*p_1990*r_1990) + sum(a_2021*p_2021*r_2021))/3 +

(sum(a_2021*p_1990*r_2021) + sum(a_2021*p_2021*r_1990))/6 -

(sum(a_1990*p_1990*r_1990) + sum(a_1990*p_2021*r_2021))/3 -

(sum(a_1990*p_1990*r_2021) + sum(a_1990*p_2021*r_1990))/6,3)

p_effect <- round((sum(a_1990*p_2021*r_1990) + sum(a_2021*p_2021*r_2021))/3 +

(sum(a_1990*p_2021*r_2021) + sum(a_2021*p_2021*r_1990))/6 -

(sum(a_1990*p_1990*r_1990) + sum(a_2021*p_1990*r_2021))/3 -

(sum(a_1990*p_1990*r_2021) + sum(a_2021*p_1990*r_1990))/6,3)

r_effect <- round((sum(a_1990*p_1990*r_2021) + sum(a_2021*p_2021*r_2021))/3 +

(sum(a_1990*p_2021*r_2021) + sum(a_2021*p_1990*r_2021))/6 -

(sum(a_1990*p_1990*r_1990) + sum(a_2021*p_2021*r_1990))/3 -

(sum(a_1990*p_2021*r_1990) + sum(a_2021*p_1990*r_1990))/6,3)

overll_differ <- round(a_effect + p_effect + r_effect,2)

a_percent <- round(a_effect/overll_differ*100,2)

p_percent <- round(p_effect/overll_differ*100,2)

r_percent <- round(r_effect/overll_differ*100,2)

#数据合并，形成一个数据集

temp <- c("Both",overll_differ,a_effect,p_effect,r_effect,a_percent,

p_percent,r_percent) %>% t() %>% as.data.frame()

#生成表头及分解分析数据集

decomposition_name<-c('sex_name','overll_difference','a_effect','p_effect','r_effect','a_percent',

'p_percent','r_percent')

names(temp) <- decomposition_name

decomposition_data<-temp

# 作图

#数据的整理，宽转长

names(decomposition_data)[2:5] <- c('Overll difference','Aging','Population','Epidemiological change')

decomposition_plot <- decomposition_data[c(1:5)]

decomposition_plot <- decomposition_plot%>%

pivot_longer(3:5, #宽数据转长数据

names_to = "varname",

values_to = "value") %>%

mutate(value=as.numeric(value)) %>%

mutate(sex_name=factor(sex_name,levels = sex_n,ordered = T))

#定义不同组颜色

my_colors <- c("#FFFB73", "#33CCCC", "#FFC773")

#画条形图

p <- ggplot(decomposition_plot, aes(x= sex_name,y=value, fill= varname)) +

geom_bar(stat="identity",position = "stack") +

coord_flip() +

#scale_fill_nejm() +

scale_fill_manual(values = my_colors) +

theme_bw()

p

decomposition_data$`Overll difference` <- as.numeric(decomposition_data$`Overll difference`)

plot <- p+ geom_point(data=decomposition_data, mapping=aes(x=sex_name,y= `Overll difference`),fill='black',color='black',size=3)

plot

###############################################################################################################################################################

###健康不平等

-----------------------

library(tidyverse)

library(data.table)

library(car)# 异方差诊断

library(MASS)# 稳健回归

library(mgcv)# 提供洛伦兹曲线拟合 (样条函数等)

library(splines)# 拟合样条函数

library(broom)

library(ggplot2)

data1<- vroom::vroom("country.csv")

data2 <- subset(data1,data1$measure_name=="DALYs (Disability-Adjusted Life Years)"&

data1$sex_name=="Both"&

data1$age_name=="All ages"&

(data1$year==1990|data1$year==2021))

# sdi 数据

sdi <- read.csv("SDI.csv",header = T,check.names = F)

sdi <- sdi %>% # 宽数据转为长数据

pivot_longer(cols = `1990`:`2021`,names_to = "year") %>%

rename(sdi=value) %>%

dplyr::select(location,year,sdi)

sdi$year <- as.integer(sdi$year)

names(sdi)[1]<-"location_name"

# 匹配 sdi 与疾病负担, 生成 data

data <- left_join(data2,sdi,by=c("location_name","year"))

# 读取人口数据

population <- data.frame()

#population<-data.frame()

for(k in 1:length(fileName)){

data = read.csv(file = paste(path,fileName[k],sep = "\\"),

header = T,stringsAsFactors = F)

population=rbind(population,data)

}

pop1 <- population %>%

dplyr::select(location_name,sex_name,age_name,year,metric_name,val)

unique(pop1$age)

pop1 <- pop1 %>%

filter(age_name=="All ages") %>%

dplyr::select("location_name","sex_name","year","val") %>%

rename(pop=val)

# 合并人口数据, 生成 mydata

mydata <- left_join(data,pop1,

by=c("location_name","sex_name","year"))

# 斜度指数的可视化 ----------------------------------------------------------------

## 1.绘图数据的准备 -----------------------------------------------------------------

# 计算总人口

a <- mydata %>%

filter(metric_name=="Number") %>%

group_by(year) %>%

summarise(sum=sum(pop))

pop1990 <- a$sum[1]

pop2021 <- a$sum[2]

# 计算加权次序

rank <- mydata %>%

mutate(pop_global=ifelse(year==1990,pop1990,pop2021)) %>%

group_by(year,metric_name) %>%

arrange(sdi) %>%

mutate(cummu=cumsum(pop)) %>% # 计算累积人口

mutate(half=pop/2) %>% # 计算该国家人口的一半

mutate(midpoint=cummu-half) %>% # 累积人口减去该国家人口一半即为人口中点

mutate(weighted_order=midpoint/pop_global) # 人口中点与总人口相比即为改国的相对位置

# 把年份设置为 factor

rank$year <- factor(rank$year)

# 选择数据

temp1 <- rank %>%

filter(metric_name=="Rate") %>%

filter(year==1990)

temp2 <- rank %>%

filter(metric_name=="Rate") %>%

filter(year==2021)

# 建模计算斜度指数

fit1 <- lm(data = temp1,val~weighted_order)

fit2 <- lm(data = temp2,val~weighted_order)

coef(fit1)

coef(fit2)

# 查看是否存在异方差（存在异方差）

ncvTest(fit1)

ncvTest(fit2)

# 使用稳健（robust）回归：重复迭代加权

r.huber1 <- rlm(data = temp1,val~weighted_order)

r.huber2 <- rlm(data = temp2,val~weighted_order)

# 获得系数与截距

coef(r.huber1)

coef(r.huber2)

# 计算稳健回归的 95% 可信区间

confint.default(r.huber1) ####这里weighted_order的系数即为斜率指数

confint.default(r.huber2) ####这里weighted_order的系数即为斜率指数

library(ggpubr)

# 2.绘图 ----------------------------------------------------------------------

color <- c("#6699FF","#990000")

colnames(rank)

p1 <- rank %>%

filter(metric_name=="Rate") %>%

ggplot(aes(x=weighted_order,y=val,fill=year,group=year,color=year))+

geom_point(aes(color=year,size=pop/1e6),alpha=0.8,shape=21)+

scale_size_area("Population\n(million)",breaks=c(200,400,600,800,1000,1200))+

geom_smooth(method = "rlm",size=0.6,alpha=0.1)+

scale_fill_manual(values = color)+

scale_color_manual(values = color)+

#增加水平虚线

geom_segment(x=0.02,xend=0.99,

y=8.254918,yend=8.254918, # coef(r.huber1)截距的位置

color="#6699FF",linetype=2,size=0.4,alpha=0.4)+

geom_segment(x=0.02,xend=0.99,

y=17.196783,yend=17.196783, # coef(r.huber2)截距的位置

color="#990000",linetype=2,size=0.4,alpha=0.4)+

# 增加某些国家的标签: 比如中国与印度

geom_text(aes(label=ifelse(location_name=="China"|location_name=="India",as.character(location_name),""),

color=year),

hjust=0,vjust=1.7,# 避免点和文字重合

size=3)+

# # 增加斜度指数标签

annotate("text",label="Slope Index of Inequality",x=1.22,y=20.228261,size=4,angle=90)+

annotate("text",label="20.23",x=1.05,y=20.228261-8.254918/2,size=3.5)+ # coef(r.huber1) 权重的系数即斜率指数

annotate("text",label="1.23",x=1.1,y=17.196783-1.234958/2,size=3.5)+ ##coef(r.huber2) 权重的系数即斜率指数

scale_x_continuous(limits = c(0,1.22),labels = c("0","0.25","0.50","0.75","1.00",""))+

xlab("Relative rank by SDI")+

ylab("Crude DALY rate (per 100,000)")+

theme_bw()

p1

# 集中指数的可视化 ----------------------------------------------------------------

# 1.绘图数据准备 ------------------------------------------------------------------

a <- mydata %>%

filter(metric_name=="Number") %>%

group_by(year) %>%

summarise(sum=sum(val))

daly1990 <- a$sum[1]

daly2021 <- a$sum[2]

ci <- rank %>%

filter(metric_name=="Number") %>%

mutate(total_daly=ifelse(year==1990,daly1990,daly2021)) %>%

group_by(year) %>%

arrange(sdi) %>%

mutate(cummu_daly=cumsum(val)) %>% # 计算累积 daly

mutate(frac_daly=cummu_daly/total_daly) %>% # 计算累积 daly 所占总体的比例

mutate(frac_population=cummu/pop_global) # 计算累积人口所占总体人口的比例

#####计算ci

# 选择数据

temp3 <- ci %>%

filter(metric_name=="Number") %>%

filter(year==1990)

temp4 <- ci %>%

filter(metric_name=="Number") %>%

filter(year==2021)

##计算集中指数

CI_1990 <- 2 * (sum(temp3$frac_daly) / nrow(temp3)) - 1

CI_2021 <- 2 * (sum(temp4$frac_daly) / nrow(temp4)) - 1

# 2.绘图 --------------------------------------------------------------------

p2 <- ci %>%

ggplot(aes(x=frac_population,y=frac_daly,fill=year,color=year,group=year))+

# 增加 X=0,y=0 两条线段

geom_segment(x=0,xend=1,

y=0,yend=0,

linetype=1,size=1,color="gray")+

geom_segment(x=1,xend=1,

y=0,yend=1,

linetype=1,size=1,color="gray")+

# 对角线

geom_segment(x=0,xend=1,

y=0,yend=1,

color="#CD853F",linetype=1,size=0.7,alpha=1)+

# 散点

geom_point(aes(fill=year,size=pop/1e6),alpha=0.75,shape=21)+

scale_fill_manual(values = color)+

scale_size_area("Population\n(million)",breaks=c(200,400,600,800,1000,1200))+

# 立方样条函数拟合洛伦兹曲线 (设置节点，边界条件)

geom_smooth(method = "gam", # 这里也可以直接用 geom_line 把点连起来

formula = y ~ ns(x,

knots = c(0.0000000001,0.25,0.5,0.75,0.9999999),# 设置节点为

Boundary.knots = c(0,1)),

linetype=1,size=0.1,alpha=0.6,se=T)+

scale_color_manual(values = color)+

# 增加两个年份的集中指数

annotate("text",label="Concentration Index",x=0.75,y=0.35,size=5)+

annotate("text",label="1990: 0.21",x=0.75,y=0.3,size=4,color="#6699FF")+

annotate("text",label="2021: 0.04",x=0.75,y=0.25,size=4,color="#990000")+

# 增加某些国家的标签，1990 年

geom_text(aes(label=ifelse(location_name=="China"&year=="1990"|location_name=="India"&year=="1990",

as.character(location_name),"")),

hjust=-0.6,vjust=0.8,

size=3)+

# 增加某些国家的标签，2021 年

geom_text(aes(label=ifelse(location_name=="China"&year=="2021"|location_name=="India"&year=="2021",

as.character(location_name),"")),

hjust=1.8,vjust=-0.0,

size=3)+

# 增加某些国家标签，人口大国

geom_text(aes(label=ifelse(location_name%in%a&year=="1990",

as.character(location_name),"")),

hjust=-0.6,vjust=0.8,

size=3)+

geom_text(aes(label=ifelse(location_name%in%a&year=="2021",

as.character(location_name),"")),

hjust=1.8,vjust=-0.0,

size=3)+

# xy 标签

xlab("Cumulative fraction of population ranked by SDI")+

ylab("Cumulative fraction of DALY")+

theme_bw()

p2

######斜率指数的年份图：

####利用上述方法计算出各个年份的SII数据

# 示例数据

sii_data <- data.frame(

year = 1990:2019,

SII = c(-120, -110, -110, -102, -100, -95, -90, -85, -80, -75,

-70, -65, -20, -50, -50, -40, -40, -35, -30, -25,

-20, -15, -10, -5, 0, 5, 10, 25, 40, 47) #####这里的SII数值是瞎编的可以行按之前的方法计算

)

# 绘制SII趋势图

ggplot(sii_data, aes(x = year, y = SII)) +

geom_point() + # 绘制数据点

geom_smooth(method = "lm", se = TRUE) + # 添加回归线和置信区间

labs(x = "Year", y = "SII") + # 设置轴标签

theme_minimal() # 使用简洁主题
